# HDAC6 inhibition alleviates mitochondrial trafficking in models of Charcot-Marie-Tooth disease type 2A

**DOI:** 10.1172/jci.insight.200106

**Published:** 2026-07-14

**Authors:** Lydia H. Jestice, Larissa Butler, Rebecca A. Lea, Kathryn I. Adamson, Jonas Van Lent, Stuart L. Johnson, Hollie Weedon, Eldriena D’Silva, Gabriele Gelezauskaite, Bob Asselbergh, Eloise Brown, Owen Laing, Christopher J. Price, Dylan Stavish, Anestis Tsakiridis, Mark O. Collins, Vincent Timmerman, Kurt J. De Vos, Alison E. Twelvetrees, Andrew J. Grierson, Ivana Barbaric

**Affiliations:** 1 School of Biosciences,; 2 Neuroscience Institute,; 3 Sheffield Institute for Translational Neuroscience, and; 4 Bateson Centre, The University of Sheffield, Sheffield, United Kingdom.; 5 Peripheral Neuropathy Research Group, Department of Biomedical Sciences, University of Antwerp, Antwerp, Belgium.; 6 Laboratory of Neuromuscular Pathology, Institute Born Bunge, Antwerp, Belgium.; 7 VIB Center for Molecular Neurology, VIB, Antwerp, Belgium.; 8 Department of Biomedical Sciences, University of Antwerp, Antwerp, Belgium.

**Keywords:** Cell biology, Neuroscience, Neuromuscular disease

## Abstract

Charcot-Marie-Tooth disease (CMT) is a group of inherited progressive conditions affecting distal motor and sensory neurons, leading to muscle weakness, pain, and loss of sensation in limbs. CMT type 2A (CMT2A) is the most common form of axonal CMT and is associated with a more severe clinical manifestation. However, there are no treatments currently available. To investigate disease mechanisms and facilitate treatment discovery, we developed an in vitro model for CMT2A by introducing the patient-specific *MFN2*^R94Q/+^ variant into human embryonic stem cells (hESCs). Isogenic variant and wild-type hESCs differentiated into spinal motor neurons with similar efficiency and gave rise to functional motor neurons in vitro. However, *MFN2*^R94Q/+^ spinal motor neurons displayed impaired mitochondrial trafficking, resulting in altered distribution of mitochondria in axons. Unbiased quantitative proteomic profiling of the endogenous MFN2 interactome revealed dose-dependent remodelling by the R94Q variant across 412 proteins, highlighting candidate mechanisms in disease pathology. Importantly, we showed that mitochondrial trafficking defects could be alleviated by treatment with an HDAC6 inhibitor. Chemical inhibition of HDAC6 also rescued the motor phenotype in a zebrafish CMT2A model. Taken together, our study reveals a variant-specific insight into CMT2A disease mechanisms and confirms HDAC6 as a promising target for further therapeutic development.

## Abstract 2

Using stem cell-derived motor neurons, we show a Charcot-Marie-Tooth 2A disease-causing variant disrupts mitochondrial transport in neurons, and a drug can reverse this defect.

## Introduction

Charcot-Marie-Tooth disease (CMT) is a group of inherited progressive conditions affecting spinal motor and sensory nerves, resulting in muscle weakness, pain, and loss of sensation in limbs of patients with CMT (1). Traditionally, CMT is classified into several different types, with the onset of some of the most severe clinical manifestations occurring within the subtype CMT2A (2, 3). CMT2A patients typically become wheelchair reliant by the age of 20 and often exhibit optic atrophy and/or hearing loss (3). CMT2A is caused by variants in Mitofusin 2 (*MFN2*), which encodes a GTPase located on the outer mitochondrial membrane (4). MFN2 has several known functions, with a canonical role of mediating the outer membrane fusion of mitochondria (5). Over 140 different variants have been mapped to *MFN2* in CMT2A patients (6–8). A particularly frequent and phenotypically severe form of the disease is caused by an autosomal dominant missense variant leading to the substitution of arginine with either glutamine or tryptophan at residue 94 of MFN2 (denoted as R94Q and R94W, respectively) (3, 7, 9). Despite substantial progress in the identification of *MFN2* variants in CMT2A patients, how such variants impact MFN2 function to give rise to the disease phenotype remains poorly understood.

CMT2A involves the progressive degeneration of spinal motor and sensory neurons, exhibiting an axonal length–dependent phenotype. An attractive hypothesis to explain the nerve length–dependent manifestation of CMT2A focuses on aberrant mitochondrial trafficking through axons (10, 11). This is supported by a seminal study in cultured rat dorsal root ganglion (DRG) neurons expressing a variant form of MFN2, which demonstrated an abnormal clustering of mitochondria in neuronal cell bodies and proximal axons as well as reduced mitochondrial mobility in variant cells (12). Subsequent studies in other in vivo and in vitro models of CMT2A, including zebrafish and mouse (13, 14), further supported the links between impaired mitochondrial trafficking and *MFN2* variants. Consequently, rescuing axonal trafficking of mitochondria has been highlighted as a promising therapeutic strategy for the treatment of CMT2A (15).

HDAC6 inhibition has been shown to effectively alleviate motor and sensory defects in a mouse model of CMT2A thought to be due to an increase in α-tubulin acetylation in peripheral nerves of variant mice (16, 17). Similar studies utilizing HDAC6 inhibition in CMT2D and CMT2F mouse models showed comparable rescues of motor and sensory deficits as well as restoring axonal transport deficits (18, 19). Moreover, HDAC6 inhibitors proved effective in alleviating mitochondrial transport in models of other neurodegenerative diseases whose pathology is also linked to mitochondrial trafficking, such as amyotrophic lateral sclerosis (20, 21). Together, these observations suggest that the HDAC6 inhibition may provide a much-needed therapeutic option for neurodegenerative diseases underpinned by mitochondrial transport defects. Nevertheless, the therapeutic efficacy of HDAC6 inhibition in the context of CMT2A requires further investigation utilizing human-based and additional physiologically relevant animal disease models.

The selective vulnerability of spinal motor neurons to CMT2A necessitates the use of disease-relevant cell types for the investigation of MFN2 disease-associated dysfunction and for testing approaches for alleviating the disease phenotype. While human spinal motor neurons are experimentally inaccessible, the advent of human pluripotent stem cell (hPSC) technology has offered a means to derive them in vitro (22, 23). hPSCs, which encompass both human embryonic stem cells (hESCs) derived from early blastocysts (24) and induced pluripotent stem cells (hiPSCs) reprogrammed from somatic cells (25), have the ability to differentiate into any cell type (24, 25). Here, we introduced the patient-specific variant *MFN2*^R94Q/+^ into hESCs to obtain isogenic variant and wild-type cells and we established a robust protocol for differentiation of hESCs into disease-relevant spinal motor neurons. We show that impaired mitochondrial trafficking and altered mitochondrial distribution are the key features of *MFN2*^R94Q/+^ motor neurons. To identify candidate molecular mechanisms underlying these defects, we performed unbiased quantitative proteomic profiling of the endogenous MFN2 interactome across isogenic *MFN2*^+/+^, *MFN2*^R94Q/+^, and *MFN2*^R94Q/R94Q^ hESCs, revealing broad and dose-dependent remodelling of the MFN2 interaction network by the R94Q variant. We further showed that mitochondrial trafficking defects in *MFN2*^R94Q/+^ motor neurons could be reversed by chemically inhibiting HDAC6. Finally, to demonstrate the in vivo relevance of these findings, we chemically inhibited HDAC6 in a zebrafish *mfn2* variant (13). This significantly rescued their aberrant swimming phenotype, thus confirming HDAC6 as a promising therapeutic target in CMT2A.

## Results

### Generation of isogenic MFN2^R94Q/+^ and MFN2^+/+^ hESCs.

**Figure 1:**
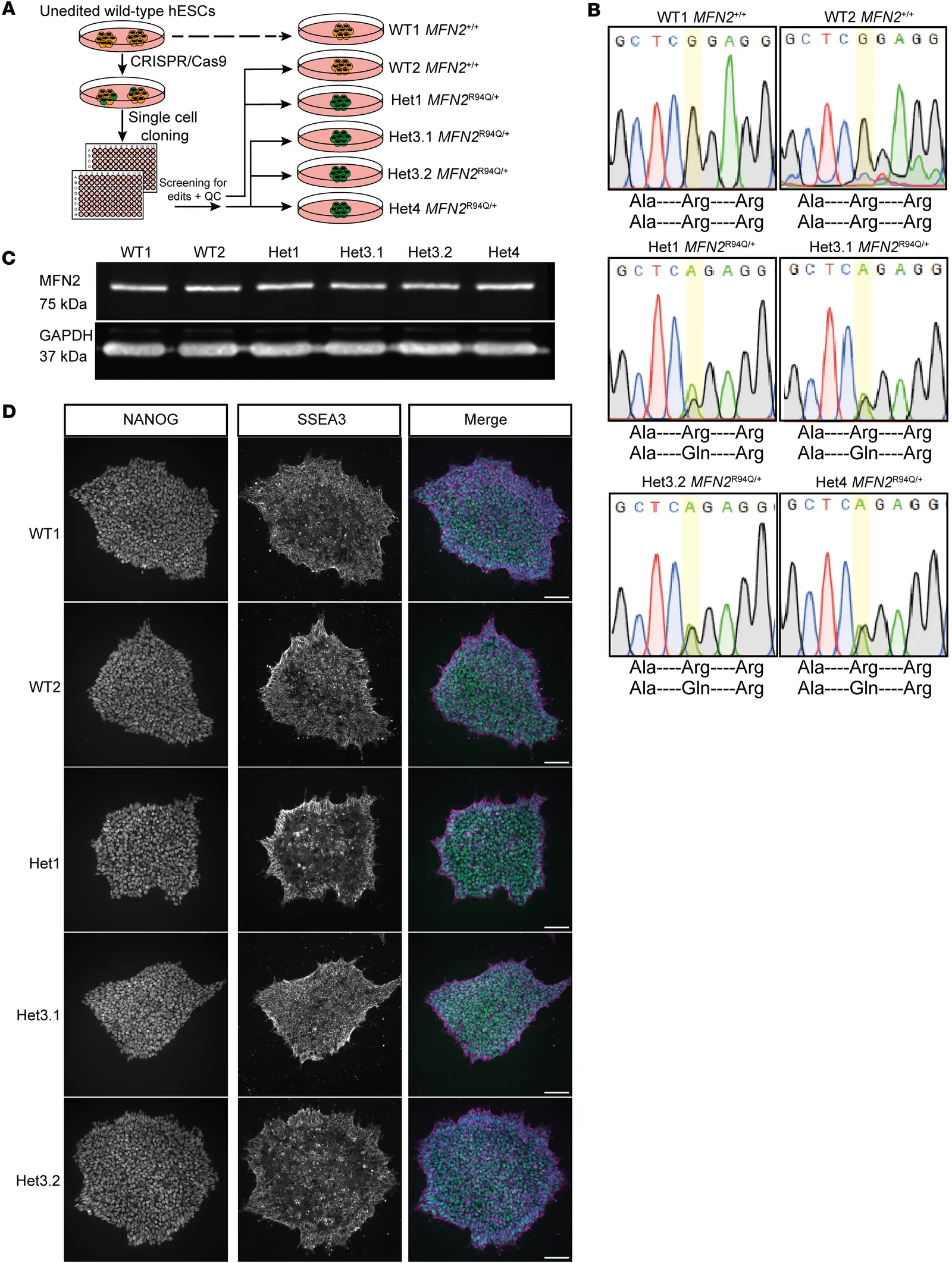
Generation of isogenic *MFN2*^R94Q/+^ and *MFN2*^+/+^ hESC clones using CRISPR/Cas9. (**A**) Schematic depicting workflow for the genome editing and cloning of edited and control sublines. Two separate arrows for clone generation indicates 2 separate CRISPR experiments. (**B**) DNA sequence analysis verified successful genome editing, with 2 peaks in the *MFN2*^R94Q/+^ clones (G and A, highlighted in yellow in the sequence) indicating heterozygous editing. Predicted amino acid sequence is shown below the sequence traces, with codon 94 in the center. (**C**) Western blot analysis of MFN2 expression in successfully edited clones. (**D**) Representative images of *MFN2*^R94Q/+^ and *MFN2*^+/+^ hESCs stained for NANOG (green) and SSEA3 (magenta) as markers of the undifferentiated hESC state. Nuclei were counterstained with Hoechst 33342. Scale bars: 100 μm.

We set out to generate a human cell–based model of CMT2A by introducing one of the most common and phenotypically severe CMT2A-causing missense variants, *MFN2*^R94Q/+^, into the hESC line MShef11 (26). To this end, we transfected euploid early-passage hESCs with a Cas9-guide RNA duplex along with a repair template designed to introduce the c.G281A substitution into the *MFN2* gene. To generate clonal lines, bulk transfected cells were single-cell sorted and expanded (Figure 1A). The resulting hESC colonies were subsequently screened for successful editing by Sanger sequencing of the target region. Following the screening, 4 heterozygous edited clones from 2 independent rounds of editing were single-cell sorted again and subclones (from herein termed Het1, Het3.1, Het3.2, and Het4) were identified, sequenced, and expanded for in-depth analyses (Figure 1B). As control cells in phenotypic assays, we used wild-type parental cells (termed WT1) and an unedited wild-type clone that had undergone CRISPR/Cas9 transfection (termed WT2) (Figure 1, A and B). All of the lines remained karyotypically diploid (Supplemental Figure 1A; supplemental material available online with this article; https://doi.org/10.1172/jci.insight.200106DS1) and did not harbor any predicted off-target variants arising during CRISPR/Cas9 mutagenesis (Supplemental Figure 1B). Introduction of the heterozygous variant did not affect MFN2 protein expression in the clones (Figure 1C). These lines also retained their undifferentiated hESC phenotype after editing, as evidenced by colony morphology (Supplemental Figure 2A) and expression of key pluripotency-associated markers (Figure 1D and Supplemental Figure 2, B and C). They also retained their pluripotency, demonstrating similar efficiency in differentiation to cells of the 3 primary germ layers to that of their parental line (Supplemental Figure 2, D and E). Overall, the CRISPR/Cas9 editing of hESCs enabled us to generate isogenic lines with and without the *MFN2*^R94Q/+^ heterozygous variant for CMT2A disease modelling.

### MFN2^R94Q/+^ hESCs efficiently differentiate into functional spinal motor neurons.

To model CMT2A in vitro, we aimed to produce spinal motor neurons from *MFN2*^R94Q/+^ hESCs. During development, limb-innervating spinal motor neurons arise in the brachial and lumbar-level spinal cord (27). They are characterized by high expression of MNX1 (HB9) (28), in addition to the expression of motor neuron markers (e.g., choline acetyltransferase [CHAT], ISLET-1/2, OLIG2, and FOXP1) (29, 30). Depending on their axial level, limb-innervating motor neurons express different HOX paralogous group (PG) genes. For example, brachial motor neurons express HOX PG 6*,* with HOX PG 5 expressed rostrally and HOX PG 8 caudally (31), whereas lumbar motor neurons express HOX PG 10 (31).

**Figure 2:**
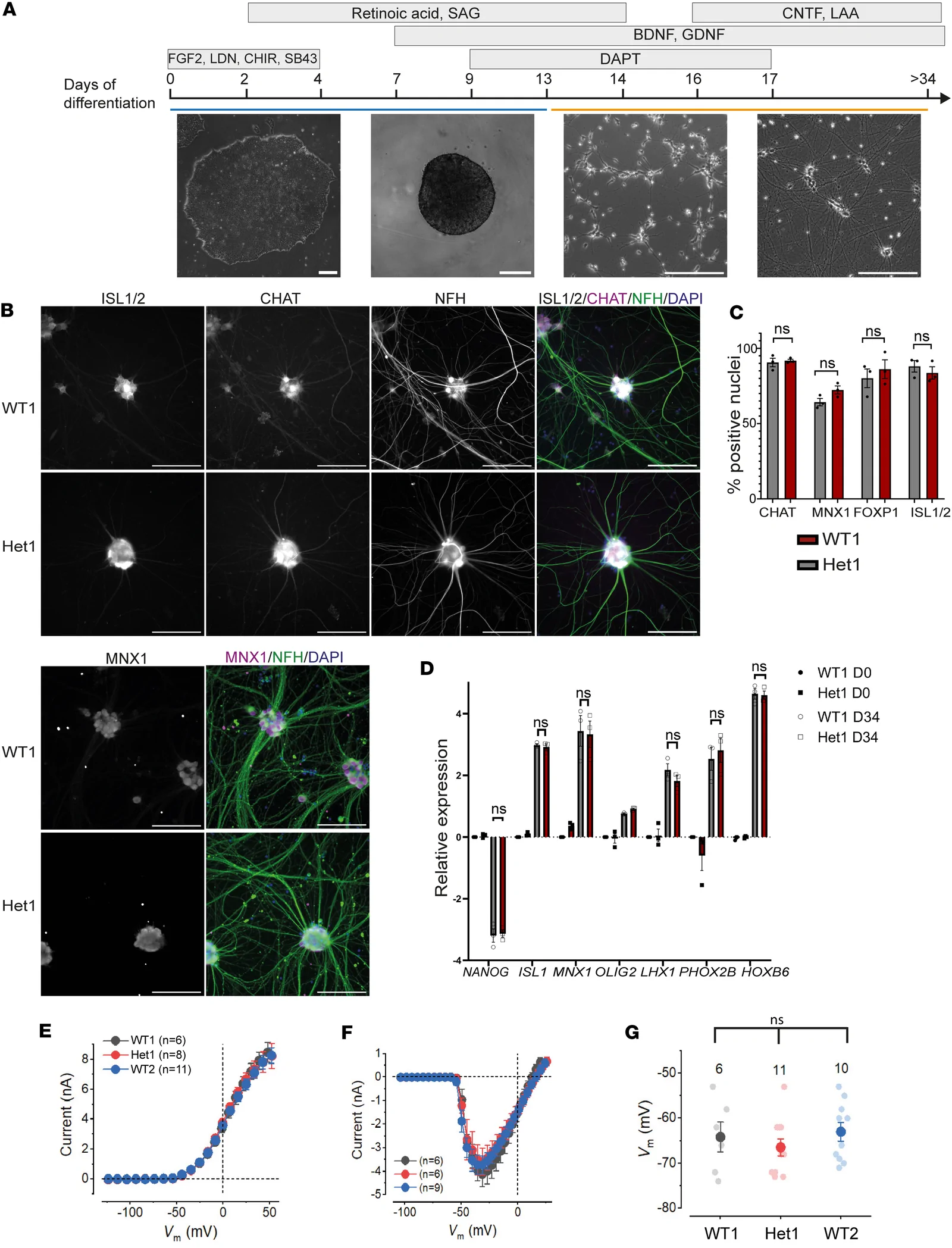
Generation of limb-innervating motor neurons from isogenic *MFN2*^R94Q/+^ and *MFN2*^+/+^ hESC clones. (**A**) Schematic depicting the differentiation protocol for the generation of spinal motor neurons from hESCs. Blue line depicts the 3D portion of the differentiation and the orange line the 2D portion. Scale bars: 100 μm. (**B**) Immunofluorescent analysis of motor neuron marker expression on day 34 of differentiation. Scale bars: 100 μm. (**C**) Quantification of immunocytochemistry for motor neuron markers (CHAT, MNX1 [HB9], FOXP1, and ISL1) on day 34 of differentiation. (**D**) Gene expression profiling by qPCR for day 34 differentiated cells, demonstrating further expression of relevant motor neuron markers (*ISL1*, *MNX1* [*HB9*], and *OLIG2*), columnar markers (*LHX1*, *PHOX2B*), and HOX markers (*HOXB6*). Data shown as the mean ± SEM of 3 biological repeats. Statistical significance was calculated with the Wilcoxon rank-sum test. NS, not significant (*P* > 0.05). (**E**) Average steady-state current-voltage (*I*-*V*) curves for *I*_K_ from all WT1 (black), Het1 (red), and WT2 (blue) neurons measured towards the end of the protocol shown in Supplemental Figure 5B. (**F**) Average peak *I*-*V* curves for *I*_Na_ from all WT1 (black), Het1 (red), and WT2 (blue) neurons measured at the peak of the inward currents shown in Supplemental Figure 5C, to isolate *I*_Na_. (**G**) Resting membrane potentials of WT1 (black), Het1 (red), and WT2 (blue) neurons measured in current clamp at 0 pA (from Supplemental Figure 5A).

Hence, to develop an efficient protocol for forelimb-innervating spinal motor neuron differentiation, we modified previously published protocols for neural differentiation from hESCs (21, 32) (Figure 2A). We then utilized the protocol to ascertain whether the *MFN2*^R94Q/+^ variant impacts the differentiation of hESCs into spinal motor neurons. We subjected *MFN2*^R94Q/+^ hESCs, alongside the *MFN2*^+/+^ WT1 and WT2 cells, to our optimized differentiation protocol (Figure 2B and Supplemental Figure 3). To compare the differentiation efficiency of *MFN2*^R94Q/+^ hESCs versus *MFN2*^+/+^ cells, we quantified the number of cells expressing different markers, including (motor) neuron markers (MNX1 [HB9], ISL1/2, CHAT) on day 34 of the differentiation protocol by immunofluorescence (Figure 2C and Supplemental Figure 4, A–D) and reverse transcription quantitative PCR (RT-qPCR) (Figure 2D and Supplemental Figure 4, E–K). All of the populations exhibited a similar overall trend in the expression of key markers of motor neuron identity and there were no significant differences in the differentiation efficiency of *MFN2*^R94Q/+^ cells compared to *MFN2*^+/+^ hESCs. Moreover, a robust expression of *HOXB5*, *HOXB6*, and *HOXB7* indicated the acquisition of a predominantly brachial identity by spinal motor neurons in all cell populations (Supplemental Figure 4, L–Q).

Finally, we assessed the functional properties of hESC-derived motor neurons on days 34 to 36 of differentiation. Motor neurons were found to be electrophysiologically active based on analysis of action potential activity and ionic currents using patch clamping, with no overt differences observed between *MFN2*^+/+^ and *MFN2*^R94Q/+^ motor neurons (Figure 2, E–G, and Supplemental Figure 5, A–C). Taken together, these data demonstrate that the *MFN2*^R94Q^ variant does not impact the differentiation efficiency or electrophysiological properties of hESC-derived spinal motor neurons on days 34–36 of differentiation.

### Altered mitochondrial morphology and distribution of mitochondria in axons of MFN2^R94Q/+^ spinal motor neurons.

**Figure 3:**
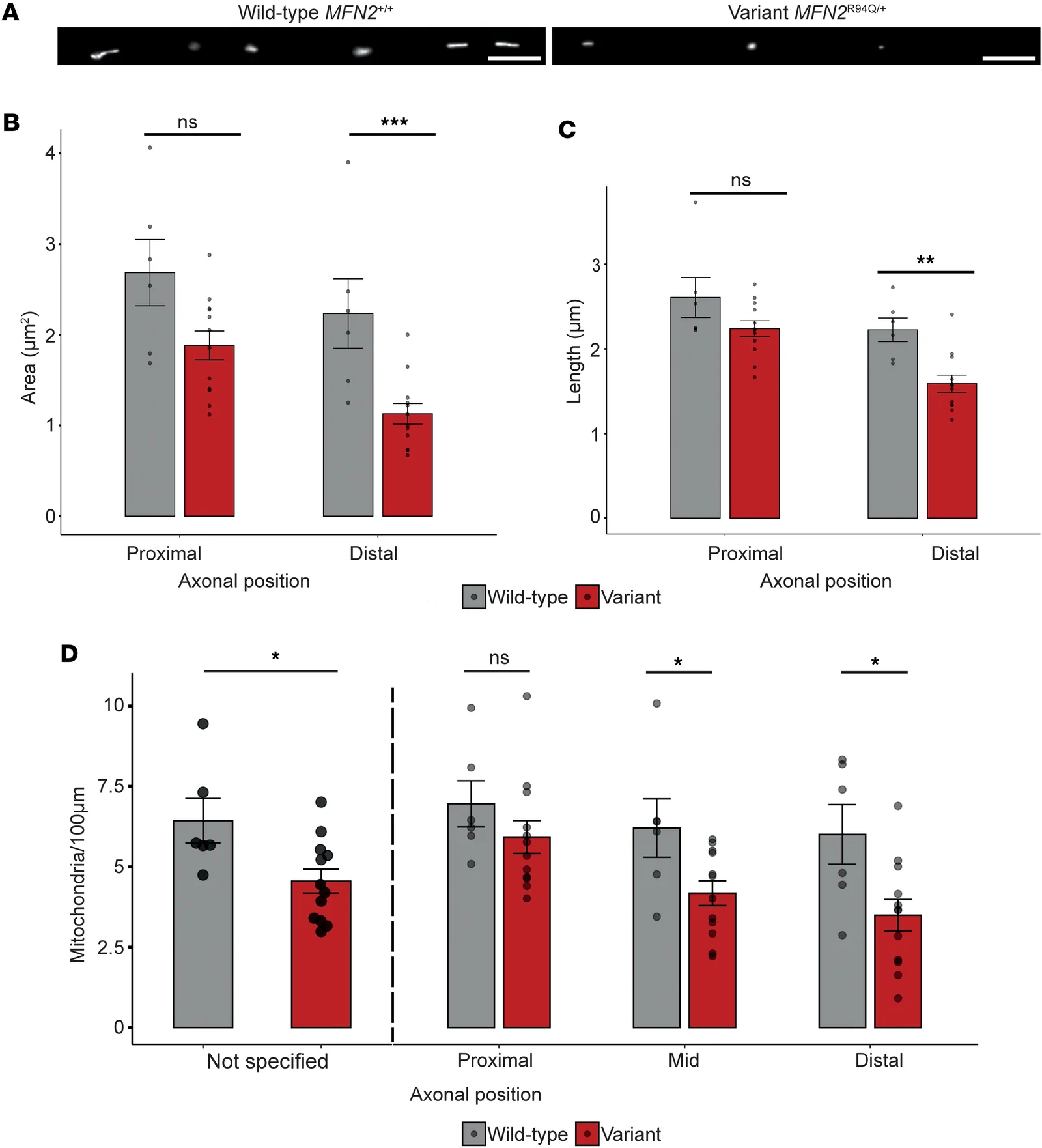
*MFN2*^R94Q/+^ disrupts mitochondrial distribution. (**A**) Representative images of Mito-DsRed2–tagged mitochondria in wild-type and variant axons after 36 days of differentiation. Scale bars: 10 μm. (**B**) Average area of mitochondria in wild-type and variant *MFN2*^R94Q/+^ hESC-derived motor neurons (day 36). Proximal regions were defined as within 500 μm from cell body, mid regions were 500 μm to 1500 μm from the cell body, and distal regions were greater than 1500 μm from the cell body of the neuron. (**C**) Average length of mitochondria in wild-type and variant *MFN2*^R94Q/+^ hESC–derived motor neurons (day 36). (**D**) Number of mitochondria per 100 μm averaged across the entire axon, and at proximal, mid, and distal axonal locations. Data shown as mean ± SEM with points representing average values from individual neurons. Statistical significance was calculated with the Wilcoxon rank-sum test (*n* = 6 wild-type, 12 variant). *P < 0.05, **P < 0.01, ***P < 0.001. NS, not significant.

Since MFN2 is critical for mitochondrial fusion, and mitochondrial morphology is directly influenced by MFN2-mediated fusion, we first examined the potential impact of the *MFN2*^R94Q/+^ variant on mitochondrial morphology. To this end, we performed high-resolution imaging of mitochondria within hESC-derived motor neurons. After 34 days of differentiation, we transfected motor neurons with a construct encoding a mitochondrial targeting sequence fused to a red fluorescent protein (Mito-DsRed2) in combination with another construct encoding enhanced green fluorescent protein (eGFP). Only a small proportion of neurons were labelled with both eGFP and Mito-DsRed2, thus enabling us to identify mitochondria within individual axons of densely populated motor neuron cultures (Supplemental Figure 6, A and B). Compared with control neurons, we observed a striking reduction in mitochondrial size in *MFN2*^R94Q/+^ neurons (Figure 3A). Quantitative analysis revealed that mitochondrial area (Figure 3B) and length (Figure 3C) were significantly decreased specifically in the distal portions of *MFN2*^R94Q/+^ neurons compared with *MFN2*^+/+^ neurons. To validate these findings, we examined an independent alternative iPSC-derived model of MFN2-related neuropathy, also carrying the R94Q variant, originating from CMT2A patient material (33). A different differentiation protocol was used in this existing iPSC line to produce ISLET-1–positive and MNX1-positive (HB9-positive) motor neurons (Supplemental Figure 6, C and D), then we imaged and quantified mitochondrial size in proximal and distal axonal zones with an alternative methodology. This independent model also showed a similar phenotype, with reduced mitochondrial size in the distal portions of *MFN2*^R94Q/+^ axons (Supplemental Figure 7, A and B).

In addition to altered mitochondrial morphology, we noted a prominent increase in the overall spacing between mitochondria in *MFN2*^R94Q/+^ motor neuron axons, as measured by the number of mitochondria per 100 μm (Figure 3D). The increased spacing between mitochondria in *MFN2*^R94Q/+^ motor neuron axons argues against a primary defect in mitochondrial fusion. Instead, this increased spacing, especially in conjunction with the more severe distal morphological changes, is consistent with impaired mitochondrial transport to distal axonal regions.

### MFN2^R94Q/+^ impairs mitochondrial trafficking in hESC-derived spinal motor neurons.

**Figure 4:**
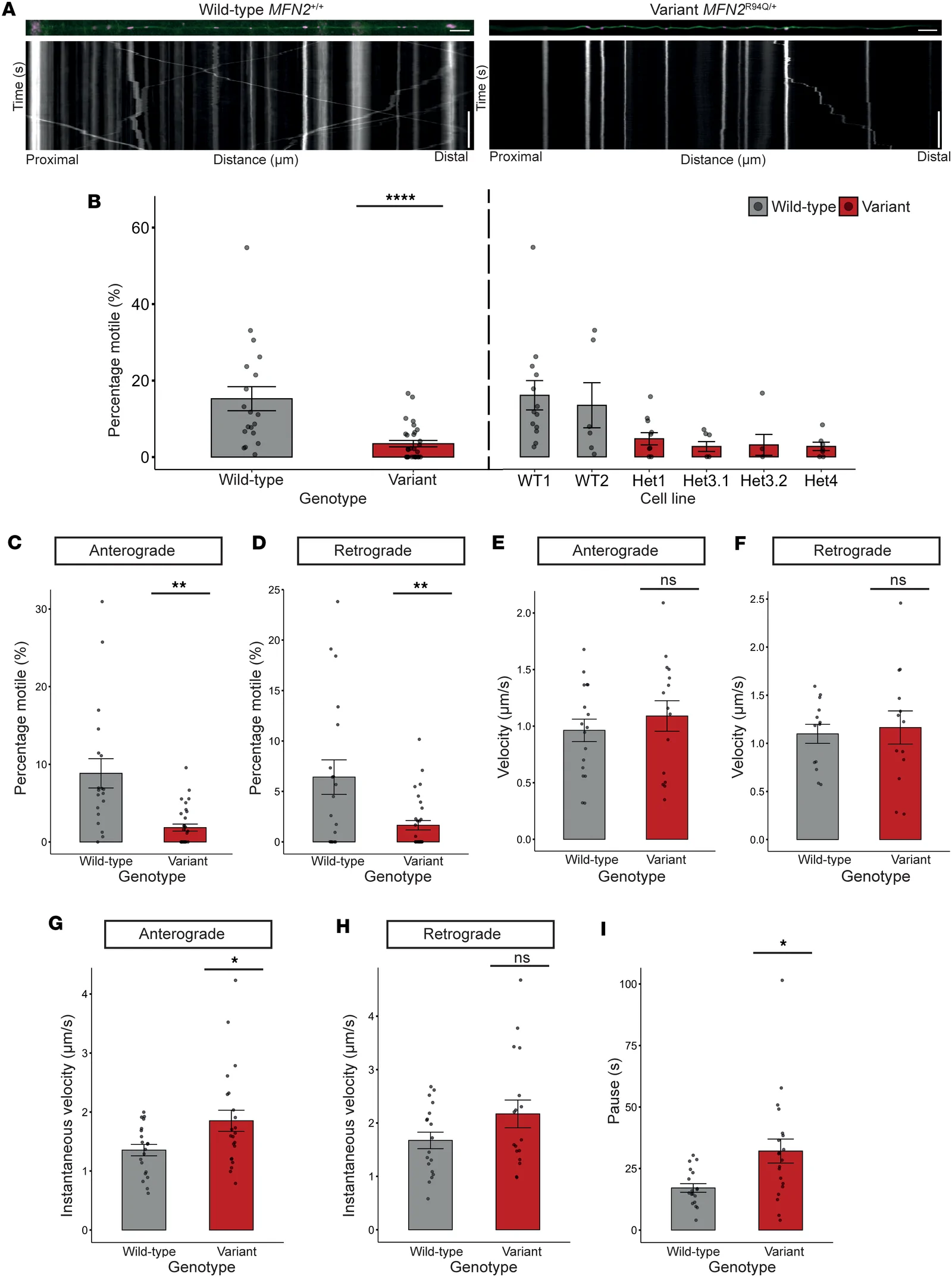
*MFN2*^R94Q/+^ reduces mitochondrial trafficking. (**A**) Representative axons and corresponding kymographs of wild-type and variant neurons after 36 days of differentiation. Stationary mitochondria are visible as straight lines on kymographs. Mitochondria in motion are shown as diagonal lines. Horizontal scale bars: 10 μm. Vertical scale bars: 100 seconds. (**B**) The percentage of motile mitochondria in control and variant lines (day 36). (**C**) Breakdown of mitochondrial movement into anterograde and (**D**) retrograde movement (day 36). (**E**) Average whole-track velocity of anterograde tracks and (**F**) retrograde tracks (day 36). (**G**) Instantaneous velocity of anterograde and (**H**) retrograde tracks (day 36). (**I**) Average length of motile track pauses measured in seconds (day 36). Data shown as mean ± SEM with points representing average values from individual neurons. Statistical significance was calculated with the Wilcoxon rank-sum test (**B**–**I**); *n* = 6 wild-type, 12 variant from 3 independent differentiations. *P < 0.05, **P < 0.01, ****P < 0.0001. NS, not significant.

MFN2 has been reported to have a role in axonal transport, with loss of protein resulting in a reduction in motile mitochondria in the axon (34). Variants in *MFN2* have also been shown to impact axonal transport (12, 33). To directly test the hypothesis that *MFN2*^R94Q/+^ impacts mitochondrial axonal transport in our model of CMT2A, we performed time-lapse microscopy of mitochondrial movement within the axons of *MFN2*^R94Q/+^ and *MFN2*^+/+^ motor neurons (Figure 4A and Supplemental videos 1 and 2). Kymographs generated from time-lapse videos were analyzed for potential differences in motility of mitochondria in *MFN2*^R94Q/+^ and *MFN2*^+/+^ motor neurons (Figure 4A and Supplemental Figure 8). We considered mitochondria motile if they moved with a speed of over 0.3 μm/s, as movements below this threshold could be attributed to actin-mediated transport (35). We found that the mitochondria in *MFN2*^R94Q/+^ hESC–derived motor neurons were significantly less motile compared with controls. Specifically, the percentage of motile mitochondria in *MFN2*^R94Q/+^ neurons was reduced to 3.5% ± 4.6% (mean ± SD) compared with 15.3% ± 13.7% in wild-type neurons (Figure 4B). This represents an approximately 77% reduction in overall mitochondrial motility in variant cells, with their transport, as measured by percentage motility, reduced in both anterograde and retrograde directions (Figure 4, C and D, and Supplemental Figure 9, A and B).

Whole-track velocity analysis of motile tracks revealed no significant difference in the average speed of motile mitochondria in either the anterograde or retrograde direction between *MFN2*^R94Q/+^ and wild-type motor neurons (Figure 4, E and F). This suggests that the overall speed of mitochondrial movement is not grossly affected by the *MFN2*^R94Q/+^ variant. Further analysis of instantaneous velocity, which reflects the speed of mitochondria at specific time points (Figure 4, G and H, and Supplemental Figure 9, C and D), revealed an increase in speed in both the anterograde and retrograde direction. This apparent discrepancy of whole-track and instantaneous velocity can be reconciled by considering the possibility of increased pausing events of mitochondria in *MFN2*^R94Q/+^ neurons. Increased pausing can indicate that mitochondria are unable to sustain continued or optimal movement speeds and is therefore associated with mitochondrial transport dysfunction. In our analysis, mitochondrial pausing was defined as a transient cessation of mitochondrial movement that lasted at least 3 frames before a mitochondrion restarted movement at a speed higher than 0.3 μm/s. The length of pauses within motile tracks was calculated for *MFN2*^+/+^ and *MFN2*^R94Q/+^, and *MFN2*^R94Q/+^ motile mitochondria exhibited longer pauses on average compared with *MFN2*^+/+^ motile mitochondria (Figure 4I). Together, these findings strongly support the notion that mitochondrial transport was more frequently interrupted in the *MFN2*^R94Q/+^ neurons, with mitochondria frequently pausing and changing direction, potentially due to impaired interactions with the motor proteins or the microtubule network.

Finally, to determine the cell-type specificity of the observed transport defects, we investigated enteric neurons derived from the same isogenic hESC lines via a vagal neural crest intermediate (36, 37) (Supplemental Figure 10A). Notably, *MFN2*^R94Q/+^ enteric neurons exhibited no significant reduction in mitochondrial motility compared to *MFN2*^+/+^ controls (Supplemental Figure 10B). These findings suggest that the mitochondrial trafficking deficit is not a generalized neuronal phenomenon but may represent a selective vulnerability of spinal motor neurons, consistent with the clinical presentation of CMT2A.

### Quantitative proteomic screening identifies candidate regulators of mitochondrial trafficking.

**Figure 5:**
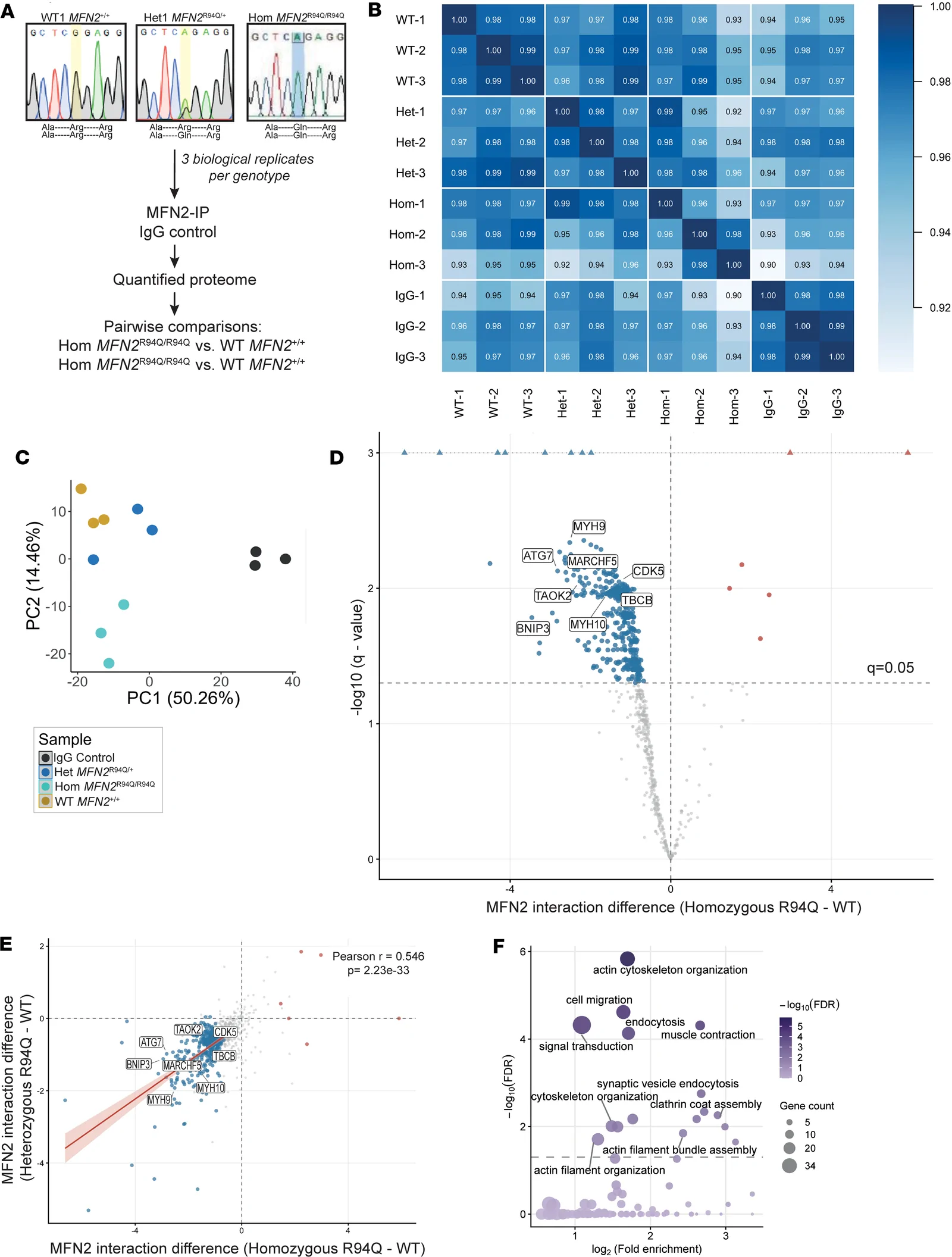
Quantitative MS identifies dose-dependent disruption of the MFN2 interactome by R94Q. (**A**) Schematic of the experimental workflow. Endogenous MFN2 was immunoprecipitated from 3 isogenic hESC lines. IgG immunoprecipitation was used as a control. Three biological replicates per genotype were analyzed by DIA-MS. (**B**) Pearson correlation matrix of log_2_-transformed protein intensities across all biological replicates demonstrates reproducibility of the MS workflow. (**C**) Principal component analysis of samples, colored by condition and genotype. Each data point represents 1 biological replicate (*n* = 3 per condition). (**D**) Bait-corrected volcano plot of *MFN2*^R94Q/R94Q^ versus *MFN2*^+/+^ MFN2 immunoprecipitations. Selected candidates referenced in the text are labelled. (**E**) Dose-dependent reduction in MFN2 partners across the isogenic allele series. Scatter plot of bait-corrected log_2_(fold change) (versus *MFN2*^+/+^) for each protein in *MFN2*^R94Q/+^ cells and *MFN2*^R94Q/R94Q^ cells. The 412 candidates significantly reduced in *MFN2*^R94Q/R94Q^ are highlighted in blue and selected candidates referenced in the text are labelled. (**F**) Gene Ontology enrichment analysis of proteins with reduced MFN2 association in *MFN2*^R94Q/R94Q^ hESCs. Bubble plot showing significantly enriched Biological Process terms for the 412 proteins with significantly reduced MFN2 association in *MFN2*^R94Q/R94Q^ relative to *MFN2*^+/+^. Enrichment analysis was performed using DAVID, with all detected proteins (4299 total) as a background. Bubble size corresponds to the number of proteins per term; color indicates the –log_10_ Benjamini-corrected FDR. Only terms with Benjamini-corrected FDR < 0.05 are shown.

To investigate the impact of the R94Q variant on the MFN2 interactome, we performed immunoprecipitation (IP) of endogenous MFN2 followed by data-independent acquisition mass spectrometry (DIA-MS) in 3 isogenic hESC lines: *MFN2*^+/+^, *MFN2*^R94Q/+^, and *MFN2*^R94Q/R94Q^ (Figure 5A). Although the homozygous R94Q variant is not observed in CMT2A patients, we reasoned that the homozygous genotype would reveal proteomic alterations that might remain below the threshold for detection in heterozygous *MFN2*^R94Q/+^ cells.

The screen quantified 4,299 protein groups, of which 644 were significantly enriched in *MFN2*^+/+^ IPs over IgG (Supplemental Table 1). Our proteomic workflow showed high reproducibility across 3 biological replicates (median Pearson’s *r* = 0.98) (Figure 5B), and principal component analysis clearly separated MFN2 IPs from IgG controls (Figure 5C). The screen recovered 16 of the 17 MFN2 interactors annotated in BioGRID (38) that were detected in our dataset (Supplemental Table 1), supporting the specificity of the immunoaffinity purification. These included MFN2’s fusion partner MFN1, the outer membrane import component SAMM50, the E3 ligase HUWE1, and components of the mitochondrial transport and tethering machinery, including RHOT2 (MIRO2) and MYO19, as well as the cytoskeletal proteins KIF14 and MYH10. Together, these data indicate that our approach efficiently captured a representative cross section of the endogenous MFN2 interactome.

Next, we investigated the impact of the R94Q variant on the MFN2 interactome by comparing proteins with differential MFN2 association across genotypes. To account for modest differences in MFN2 recovery across biological replicates, all values were bait-corrected (Supplemental Table 1). Pairwise comparison of *MFN2*^R94Q/R94Q^ versus *MFN2*^+/+^ identified 412 proteins with significantly reduced MFN2 association and 6 candidates with significantly increased association (Figure 5D). While the *MFN2*^R94Q/+^ versus *MFN2*^+/+^ comparison was underpowered at the single-protein level, the proteins with reduced MFN2 association showed a clear dose-dependent pattern across the isogenic allele series, consistent with the simultaneous co-purification of wild-type and R94Q MFN2 binding partners from heterozygous lysates. Specifically, 410 out of 412 candidates with reduced MFN2 association in *MFN2*^R94Q/R94Q^ cells also showed a matching downward trend in *MFN2*^R94Q/+^ cells (Figure 5E and Supplemental Table 1).

To assess the biological relevance of these candidates, we searched the literature for known functions of the proteins with reduced MFN2 association. We note that unbiased co-IP proteomics will inevitably identify proteins that are unlikely to interact with MFN2 in a biologically meaningful context, including nuclear and transcriptional machinery components whose subcellular localization is incompatible with direct interaction with an outer mitochondrial membrane protein. We therefore focused our interpretation on candidates with subcellular localizations and known functions compatible with MFN2 biology, with the full dataset provided in Supplemental Table 1 as a resource for future investigation. Among the most notable individual hits with reduced interaction were MARCHF5, a mitochondrial outer-membrane E3 ligase that regulates MFN2 turnover and ER-mitochondria contacts (39), ATG7, an E1-like autophagy enzyme required for autophagosome biogenesis (40, 41), and BNIP3, a mammalian Atg32 homolog that mediates mitophagy (42), suggesting a potential disruption to mitochondrial quality control in variant cells. We also noted reduced association of factors with reported roles in axonal transport and cytoskeletal regulation, including CDK5, a kinase that modulates axonal transport of vesicles and organelles (43, 44), TAOK2, which coordinates ER-microtubule tethering (45), and TBCB, a tubulin-specific chaperone that regulates microtubule dynamics (46).

Beyond individual candidates, Gene Ontology enrichment analysis of the 412 proteins with reduced MFN2 association in homozygous R94Q cells, performed against a background of all proteins detected in the MS experiment, identified actin cytoskeleton organization as the most significantly enriched biological process term (34 proteins, FDR = 1.47 × 10^–6^), followed by cell migration (30 proteins, FDR = 2.42 × 10^–5^) and endocytosis (25 proteins, FDR = 7.39 × 10^–5^) (Figure 5F). The actin cytoskeleton organization term was driven by a functionally diverse set of proteins, including cytoskeletal regulators with established roles in axonal biology (CDK5, TMOD3, VASP, and COBL), Rho GTPase signaling components (CDC42, ARHGEF17, ARHGAP8, and FMNL2), and the non-muscle myosin heavy chains MYH9 and MYH10. These findings raise the possibility that the R94Q variant disrupts MFN2 association with actin cytoskeletal regulatory machinery, potentially impairing actin-based mitochondrial anchoring in addition to microtubule-dependent transport, although validation in a neuronal context will be required to establish the pathological relevance of these interactions. Collectively, these data reveal that the R94Q substitution broadly remodels the MFN2 interactome and provide a hypothesis-generating resource for future mechanistic investigation of how the *MFN2*^R94Q/+^ variant drives selective vulnerability in motor neurons.

### HDAC6 inhibition rescues tubulin acetylation and improves mitochondrial trafficking in MFN2^R94Q/+^ hESC–derived spinal motor neurons.

Several studies pointed to HDAC6 inhibition as a promising approach to alleviating mitochondrial trafficking defects (18, 21, 47). However, the efficacy of HDAC6 inhibition in alleviating trafficking impairment underpinned by *MFN2*^R94Q^ in human neurons remains unknown. Here, we utilized our hESC-derived *MFN2*^R94Q/+^ spinal motor neurons to test the effect of a selective HDAC6 inhibitor, ACY-738 (48), on mitochondrial trafficking within axons.

**Figure 6:**
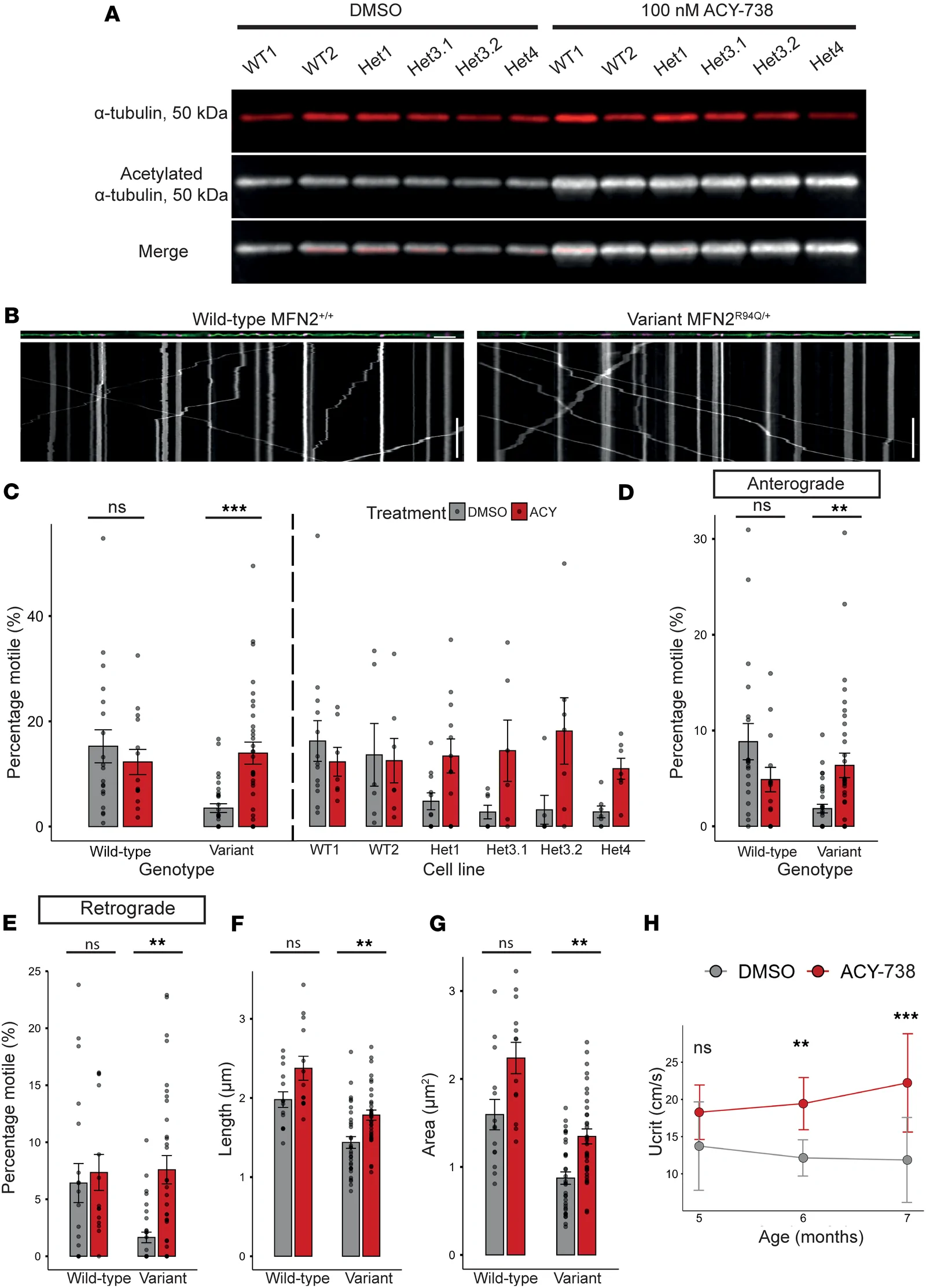
HDAC6 inhibition by ACY-738 partially rescues mitochondrial trafficking defect in axons of *MFN2*^R94Q/+^ motor neurons and motor deficits in MFN2 variant zebrafish. (**A**) Western blot analysis of acetylated α-tubulin in day 35 hESC–derived motor neurons treated for 24 hours with either DMSO or 100 nM ACY-738. (**B**) Representative axon and corresponding kymographs of day 36 wild-type and variant neurons after 24 hours of 100 nM ACY-738 treatment. Horizontal scale bars: 10 μm. Vertical scale bars: 100 seconds. (**C**) The percentage of motile mitochondria in DMSO- or ACY-738–treated control and variant lines. (**D**) Percentage of anterograde mitochondria and (**E**) retrograde mitochondria in DMSO- or ACY-738–treated wild-type or variant lines. (**F**) Average length and area (**G**) of mitochondria in wild-type and variant *MFN2*^R94Q/+^ hESC–derived motor neurons treated with DMSO or 100 nM ACY-738. Data shown as mean ± SEM with points representing average values from individual neurons in **C**–**G**. Statistical significance was calculated with a Mann-Whitney *U* test (**C** to **E**); *n* = 6 wild-type, 12 variant from 3 independent differentiations. **P < 0.01, ***P < 0.001. NS, not significant. (**H**) The change in critical swimming velocity (U_crit_) of *mfn2*^hu3528/hu3528^ zebrafish between 5 and 7 months after fertilization following intermittent treatment by combined oral and immersion dosing with vehicle (1% DMSO) or ACY-738. Points and error bars represent mean and standard deviations of U_crit_ of individual fish measured at each time point (*n* = 8 vehicle-treated, *n* = 11 ACY-738–treated fish at 5 and 6 months after fertilization; *n* = 7 vehicle-treated, *n* = 11 ACY-738–treated fish at 7 months after fertilization). NS, not significant. **P < 0.01; ***P < 0.001 by 2-way ANOVA with Dunnett’s T3 multiple-comparison test between treatment groups at each time point.

*MFN2*^R94Q/+^ hESC–derived motor neurons and their *MFN2*^+/+^ counterparts were transfected with the Mito-DsRed2 plasmid and the eGFP plasmid and were treated for 24 hours with 100 nM ACY-738. We confirmed that the ACY-738 treatment increased the amount of acetylated α-tubulin in treated cells (Figure 6A). The effect of treatment on mitochondrial transport was determined by imaging of DsRed2-labelled mitochondria by time-lapse microscopy. Strikingly, tracking of mitochondria revealed an increase in the percentage of motile mitochondria in ACY-738–treated *MFN2*^R94Q/+^ cells (Figure 6, B and C). The effect of ACY-738 treatment was apparent for both anterograde (Figure 6D and Supplemental Figure 11A) and retrograde (Figure 6E and Supplemental Figure 11B) movement of mitochondria within *MFN2*^R94Q/+^ axons. Furthermore, the length and area of *MFN2*^R94Q/+^ mitochondria in the distal portions of the axons were significantly increased following treatment (Figure 6, F and G), suggesting that restored trafficking facilitates distal fusion events. To ensure these effects were not confounded by cytotoxicity, we quantified the apoptotic marker cleaved caspase-3. No significant induction of apoptosis was observed (Supplemental Figure 12, A–C), indicating that the dose is well tolerated.

To verify that these improvements were driven by α-tubulin acetylation, we targeted an alternative deacetylase using the SIRT2 inhibitor, AGK2 (49). Similar to HDAC6 inhibition, SIRT2 inhibition increased α-tubulin acetylation (Supplemental Figure 13A), and the percentage of motile mitochondria in *MFN2*^R94Q/+^ motor neurons (Supplemental Figure 13B), further supporting the role of microtubule acetylation in facilitating axonal transport in this context. Together, these data demonstrate that pharmacological modulation of microtubule acetylation can bypass transport defects in CMT2A motor neurons.

### Chemical inhibition of HDAC6 rescues motor defects in MFN2 variant zebrafish.

To investigate whether the potential beneficial effect of HDAC6 inhibition in cultured *MFN2*^R94Q/+^ human motor neurons translates to the more complex physiological situation in vivo, we utilized a zebrafish CMT2A model. We previously showed that *mfn2* variant zebrafish carrying a null allele show a progressive motor phenotype associated with impaired mitochondrial transport and degeneration of the distal axon (13). We performed *mfn2*^hu3528/+^ in-crosses and randomly separated the offspring into 2 treatment groups that were housed in separate tanks that were not connected to the recirculating water system in the aquarium. From 8 days after fertilization, the control group was accommodated in tank water containing 2% DMSO and the treatment group was housed similarly, but the tank water also contained 300 nM ACY-738. To minimize the possibility of off-target and toxic effects, an intermittent dosing strategy was used, alternating ACY-738/vehicle–treated water with untreated water on a biweekly basis. Despite these modifications, we noticed that the zebrafish housed in these tanks developed at a much slower rate than those maintained on the recirculating aquarium system.

Oral dosing of vehicle or ACY-738 was commenced in the diet from 80 days after fertilization, and was also administered on an intermittent basis, alternating with untreated food on the same biweekly schedule as the immersion treatment. The vehicle treatment group was fed 16 mg of Gelly Belly diet twice per day, and the treatment group was fed 16 mg of Gelly Belly diet containing 0.058% (w/w) ACY-738 twice per day. To determine effects of treatment on motor function, we investigated the critical swimming velocity (U_crit_) of ACY-738– and vehicle-treated fish at monthly intervals between 5 and 7 months after fertilization, with U_crit_ calculated for both treatment groups at each time point. From 6 months, the U_crit_ of ACY-738–treated fish was significantly increased compared with the *mfn2*^hu3528/hu3528^ variants, indicating that ACY-738 treatment improved the defective swimming phenotype (Figure 6H). At 7 months, the experiment was terminated on welfare grounds, as the vehicle-treated animals were showing signs of distress. This result shows that ACY-738 treatment restores defective swimming in a zebrafish CMT2A model.

## Discussion

To date, there are no approved treatments for CMT2A. Further mechanistic understanding of the disease is required to identify targets that may alleviate disease symptoms. Reliable disease models that recapitulate key features of the disease phenotype are critical for deciphering disease pathophysiology and uncovering therapeutic options. In this study, we capitalized on hESC technology to establish an isogenic human cell–based model of CMT2A. To this end, we combined CRISPR/Cas9 genome editing and directed differentiation of hESCs to derive isogenic spinal motor neurons harboring one of the most prevalent and phenotypically severe CMT2A-causing variants, *MFN2*^R94Q/+^. We then utilized this cell-based model to analyze CMT2A disease mechanism and test a pharmacological approach to alleviating the disease phenotype.

Our findings demonstrate that *MFN2*^R94Q/+^ motor neurons exhibit significant defects in mitochondrial transport. Specifically, we observed a reduction in the percentage of motile mitochondria compared with wild-type controls paired with an axonal location–dependent reduction in mitochondrial size, indicating impaired mitochondrial trafficking. This finding is consistent with previous observations in a complementary hiPSC-based model of CMT2 (33), which we also further analyzed in our study (Supplemental Figure 7, A and B). A significant strength of our approach is the use of an isogenic hESC-based system, which isolates the pathogenic effects of the MFN2 R94Q variant from the potentially confounding influence of individual genetic backgrounds or line-to-line variations. To ensure that our findings were a true reflection of the variant effect and not unique to a single genomic context, we cross-validated our observations in an independent, patient-derived hiPSC model carrying the same R94Q variant (Supplemental Figure 7). The consistent observation of mitochondrial phenotypes across these 2 models — despite the use of different differentiation protocols and distinct genetic origins — underscores the robustness of mitochondrial transport impairment as a hallmark of MFN2 R94Q pathology. Our dual-model approach demonstrated clear consistency between engineered hESCs and patient hiPSCs, reinforcing the physiological relevance of our findings and suggesting that the R94Q variant in these cells exerts a pervasive effect on mitochondrial dynamics irrespective of individual genetic background.

To dissect the underlying mechanisms of mitochondrial transport impairment, we analyzed mitochondrial motility dynamics in detail. Whole-track velocity was unchanged by *MFN2*^R94Q/+^ but *MFN2*^R94Q/+^ mitochondria spent more time paused. We explained this discrepancy by noting that *MFN2*^R94Q/+^ mitochondria had increased instantaneous velocity, such that faster movements with more pauses averaged out to a similar whole-track velocity to that of *MFN2*^+/+^ neurons. The pausing of transport could be attributed to several different causes, including altered intracellular calcium concentrations (50), motor protein dissociation from the microtubules (51), or motor protein dissociation from the intracellular cargo of interest (52). To our knowledge, this is the first model to show increases in anterograde instantaneous velocity of mitochondria in the presence of a CMT2A-causing variant, and this increase may be attributed to increased kinesin-1 processivity or a greater contribution of other kinesin motor proteins, such as kinesin-3, which can achieve greater speeds than kinesin-1 (53, 54). Taken together, this analysis of transport dynamics is suggestive of a disruption to the ability of motor proteins to carry out their function efficiently.

To investigate the biochemical basis of this disrupted motor function, we performed quantitative IP combined with MS on MFN2’s endogenous interactome across the isogenic allele series. Our analysis identified 412 partners with significantly reduced MFN2 association in *MFN2*^R94Q/R94Q^ cells. The majority of candidates showed concordant downward trends in heterozygous *MFN2*^R94Q/+^ cells, indicating dose-dependent disruption of the MFN2 interactome. Among the candidates with reduced MFN2 association, several are of particular functional relevance. MARCHF5, a mitochondrial outer-membrane E3 ubiquitin ligase that regulates MFN2 turnover and ER-mitochondria tethering, modulates the local pool of MFN2 available for adapter engagement (39). CDK5 is a known regulator of axonal transport (43, 44), providing a plausible biochemical basis for the altered motor engagement implied by our motility analysis. Additionally, the mitophagy machinery components ATG7 (40, 41) and BNIP3 (42) were among the reduced interactors, raising the possibility that variant-driven interactome remodelling impairs not only mitochondrial transport but also the quality control mechanisms responsible for clearing dysfunctional mitochondria from axons, a process of particular importance in the distal regions of long motor neuron axons. Gene Ontology enrichment analysis of the 412 reduced interactors independently identified actin cytoskeleton organization as the most significantly enriched biological process term. These findings raise the possibility that the R94Q variant disrupts MFN2 association with actin cytoskeletal regulatory machinery. Given the multiple roles of actin in mitochondrial biology, including anchoring, fission, and fusion, the functional consequences of this disruption may extend beyond trafficking, although validation in a neuronal context will be required to establish the pathological relevance of these interactions. Taken together, the dose-dependent loss of interactions spanning MFN2’s regulatory, transport, cytoskeletal, and quality control networks supports a model in which the R94Q substitution broadly destabilizes the MFN2 interactome in a manner consistent with the mitochondrial trafficking defects observed in MFN2^R94Q/+^ motor neurons. We emphasize that this dataset identified candidate mechanisms rather than established causal pathways, and prioritized follow-up of identified candidates will be required to determine which specific interactome changes drive the observed trafficking deficits.

A defining feature of CMT2A is the selective vulnerability of long-axon motor neurons despite the ubiquitous expression of MFN2. To investigate whether the trafficking defect we observed reflects a generalized neuronal phenomenon or a lineage-restricted vulnerability, we differentiated our isogenic hESC lines into enteric neurons, a population that is not known to be involved in CMT2A clinical manifestation. *MFN2*^R94Q/+^ enteric neurons differentiated efficiently and maintained mitochondrial motility levels comparable to wild-type controls (Supplemental Figure 10, A and B). The selective vulnerability of spinal motor neurons in our model therefore mirrors the clinical presentation of CMT2A, suggesting that the R94Q variant interacts with cell-type-specific factors, potentially including the unique metabolic demands, axonal geometry, or cytoskeletal organization of long-projection motor neurons, to produce its pathogenic effect.

Finding a drug that rescues an aberrant motor neuron phenotype could provide a platform for developing pharmacological treatments for CMT2A patients. Based on our identification of aberrant mitochondrial trafficking as a key phenotype in CMT2A human motor neurons, we focused on finding a drug that could revert this aberration. Mitochondrial axonal transport can be increased by treatment of neurons with HDAC6 inhibitors, which are thought to mediate their effects on mitochondrial trafficking through increasing acetylation of tubulin (19, 21, 47, 55). No loss of acetylation was noted in untreated *MFN2*^R94Q/+^ motor neurons, whereas other murine-based models in the literature have shown disruption to tubulin acetylation in the presence of CMT2A-associated variants (16, 56). Using an HDAC6 inhibitor, ACY-738, we showed a partial reversion of the diseased phenotype, as ACY-738–treated *MFN2*^R94Q/+^ cells exhibited increased percentage of motile mitochondria compared with non-treated *MFN2*^R94Q/+^ motor neurons, and mitochondrial length and area in the distal axon were significantly increased following treatment, suggesting that restored trafficking enables the distal fusion events necessary to maintain healthy organelle morphology. To confirm whether this effect was clinically relevant, we tested ACY-738 in a zebrafish CMT2A model with loss of function of mfn2, where it significantly alleviated motor phenotypic defects. The convergence of rescue effects across a human cell–based model and an in vivo vertebrate system strengthens confidence in HDAC6 inhibition as a promising therapeutic strategy for CMT2A and supports its progression towards preclinical development. Importantly, SIRT2 inhibition also restored mitochondrial motility in *MFN2*^R94Q/+^ motor neurons, confirming that the rescue effect is attributable to increased tubulin acetylation rather than an off-target effect of ACY-738 specifically.

Several limitations of the present study should be acknowledged. First, while HDAC6 inhibition with ACY-738 partially restored mitochondrial motility in *MFN2*^R94Q/+^ motor neurons, the rescue was incomplete, and the long-term functional consequences of this treatment on neuronal survival, axonal integrity, and synaptic activity were not investigated. These represent important outcomes for future preclinical assessment, alongside a comprehensive long-term safety profiling of ACY-738, which extends beyond the scope of the present study. Second, while our data establish impaired mitochondrial trafficking as a prominent and reproducible phenotype in *MFN2*^R94Q/+^ motor neurons, whether this represents the primary pathogenic driver or a secondary consequence of broader mitochondrial dysfunction remains an open question that cannot be resolved from the current dataset alone. Addressing this issue will require longitudinal studies integrating additional functional readouts, including ATP production, mitochondrial membrane potential, calcium buffering capacity, and markers of axonal degeneration. Third, the zebrafish rescue experiments employed an *mfn2*-null model rather than the specific R94Q variant, limiting direct genotypic equivalence with the human in vitro model. We note, however, that genotype-specific evidence is provided by our isogenic hESC system, whereas the zebrafish experiments provide complementary in vivo proof of concept for the therapeutic approach. Fourth, the selective vulnerability of spinal motor neurons was inferred from a comparison with enteric neurons, a population not associated with the clinical manifestations of CMT2A. While this comparison provides initial evidence for lineage-restricted vulnerability, the evaluation of additional neuronal subtypes will be required to strengthen this conclusion. Finally, our proteomic data identified disruption of MFN2 association with the actin cytoskeletal regulatory machinery as a candidate pathological mechanism; however, we acknowledge that this conclusion is speculative in the absence of direct experimental validation of actin-dependent mitochondrial anchoring or transport in a neuronal context, and that the enrichment of actomyosin components may partly reflect the hESC cellular context in which the screen was performed. Together, these limitations define a clear agenda for future investigation building on the core findings of this study, which establish impaired mitochondrial trafficking as a defining feature of *MFN2*^R94Q/+^ motor neurons and identify HDAC6 inhibition as a promising therapeutic strategy warranting further development.

## Methods

### Sex as a biological variable.

Zebrafish sex determination happens at 20–30 days after fertilization, and sex can be determined using externally visible phenotypes by 3–4 months of age. The zebrafish used in this study were sexed and genotyped at 4 months of age, and at this stage sex- and treatment-matched experimental groups for behavioral analysis were determined.

### hPSC maintenance.

The hESC line used in this study was the MShef11 hESC line (RRID: CVCL_BW83) (The University of Sheffield) (26). A working bank of MShef11 was made from cells around 20 passages from the original derivation. In this study, cells were thawed from a working bank and were grown for no longer than 10 passages after thawing. Cells were grown in Essential 8 (E8) (made in-house based on Chen et al., ref. 57) on GelTrex (Life Technologies, A1413202), and were maintained at 37°C under a humidified atmosphere of 5% CO_2_ in air. Cells were cultured in the absence of antibiotics and tested for mycoplasma quarterly. DNA of cells from the working bank was analyzed for karyotypic abnormalities by low-pass whole genome sequencing. After 10 passages in culture, cells were checked by qPCR for presence of the most common culture-acquired karyotypic abnormalities (1q, 12p, 17q, and 20q), as previously described (58, 59).

### Zebrafish.

All zebrafish (The University of Sheffield) were maintained in the University of Sheffield Bateson Centre Aquaria at 28°C. *mfn2*^hu3528/+^ zebrafish have been previously described (13). To generate experimental cohorts, *mfn2*^hu3528/+^ fish were in-crossed, and *mfn2*^hu3528/hu3528^ fish were identified using PCR-based genotyping according to Chapman et al. (13).

For drug dosing studies, zebrafish were housed in 3.5 L tanks from 5 days to 6 months old at a maximum stocking density of 10 fish per liter. Beginning at 8 days old, ACY-738/2% DMSO (treatment) or 2% DMSO (vehicle) was given intermittently. Treatment or vehicle was applied for 3.5 days and then withdrawn for 3.5 days, to reduce the chances of toxicity. For oral dosing, ACY-738 was also formulated in Gelly Belly food mix (Florida Aqua Farms) according to a previously described method (60). In preliminary studies, we established that a 3.5 L tank of zebrafish, at a stocking density of 10 fish per liter, would eat at most 16 mg of Gelly Belly diet twice per day. Thus, ACY-738 was formulated at a dose of 0.058% (w/w) in Gelly Belly and 16 mg of this was fed to each 3.5 L tank twice per day. The vehicle group received Gelly Belly diet without ACY-738. Swimming endurance was measured using a custom-built swim tunnel apparatus and critical swimming speed (U_crit_) was calculated as previously described (13).

### CRISPR/Cas9 genome editing.

Guides and repair template were designed for exon 3 of *MFN2* using the Horizon CRISPR design tool (https://horizondiscovery.com/products/tools/CRISPR-Targeted-Gene-Designer) (cRNA sequence: TCAGTGAGGTGCTGGCTCGG Repair Template: AAGTGAGAGGCATCAGTGAGGTGCTGGCTCAGAGGCACATGAAAGTGGCTTTTTTTGGCCG). cRNA and ALT-R CRISPR-Cas9 tracrRNA (IDT, 1072532) were mixed in equimolar concentrations and heated to 95°C for 5 minutes. The MShef11 cells were transfected with HiFi Cas9 Nuclease V3 (IDT), cRNA:tracrRNA complex, and repair template using a DigitalBio Microporator (Thermo Fisher Scientific) with the following settings: 1400 V, 20 ms, 1 pulse. Cells were grown in E8 supplemented with 10 μM Y-27632 for 48 hours. Cells were then single-cell sorted using the FACS Jazz (BD Biosciences) into GelTrex-coated 96-well plates. After approximately 14 days of growth, a third of the well contents was transferred to the new culture vessel for expansion for cryopreservation. The rest of the sample was taken for DNA analysis. Sanger sequencing was performed to detect clones with the correct genome editing of *MFN2*. Putative off-target effects were detected in silico using the IDT Guide Checker (https://eu.idtdna.com/site/order/designtool/index/CRISPR_SEQUENCE). The top 5 off-target locations (Supplemental Table 2) were checked by Sanger sequencing. Four heterozygous edited clones, *MFN2*^R94Q/+^ Het1 (RRID: CVCL_E3QL), *MFN2*^R94Q/+^ Het3.1 (RRID: CVCL_E3QM), *MFN2*^R94Q/+^ Het3.2 (RRID: CVCL_E3QN), and *MFN2*^R94Q/+^ Het4 (RRID: CVCL_E3QP) were derived in independent CRISPR/Cas9 experiments. *MFN2*^+/+^ WT2 clone (RRID: CVCL_E3QK) and MFN2 ^R94Q/R94Q^ Hom1 (RRIDL CVCL_ F4CS) were derived from the same targeting/cloning experiment as *MFN2*^R94Q/+^ Het1.

### RNA extraction, reverse transcription, and qPCR.

For qPCR analyses, RNA was extracted using the Norgen Total RNA Purification Plus Kit (Norgen). RNA was reverse transcribed using High-Capacity Reverse Transcriptase (Applied Biosystems, 4368813) according to the manufacturer’s instructions. Each 10 μL qPCR reaction contained 1× PowerTrack SYBR Green Master Mix (Thermo Fisher Scientific, 46109), 100 nM of each forward and reverse primers (Supplemental Table 3), and 10 ng of RNA. The PCR reaction was run on a QuantStudio 12K Flex Thermocycler (Thermo Fisher Scientific) with the following parameters: 50°C for 2 minutes, 95°C for 10 minutes, then 40 cycles of 95°C for 15 seconds, 60°C for 1 minute. The cycle times were obtained from the QuantStudio 12K Flex Software with auto baseline settings and were then exported to Microsoft Excel for analysis using the ΔΔCt method.

### Immunocytochemistry.

Cells were fixed with 4% paraformaldehyde (PFA) for 15 minutes at room temperature. Cells were then blocked and permeabilized with 0.2% Triton X-100 (Sigma-Aldrich) in PBS supplemented with 10% fetal calf serum for 1 hour at room temperature. Primary antibodies (Supplemental Table 4) were incubated with samples at 4°C overnight. Secondary antibodies (Supplemental Table 5) were incubated for 1 hour at room temperature in the dark. Nuclei were counterstained with Hoechst 33342 (Thermo Fisher Scientific). Stained cells were imaged using either the Incell Analyser 2000 (GE Healthcare) or LSM 880 AiryScan Confocal (Zeiss). Images were analyzed using Cell Profiler (61) using custom-made protocols.

### Differentiation of hESCs into spinal motor neurons.

Motor neuron differentiation was optimized based on the protocols published by Maury et al. (32) and Guo et al. (21). hPSCs (3000 cells) were plated on day 0 in 96-well U-bottomed low-attachment plates (Greiner) in N2B27 media (DMEM-F12 50:50 mix with Neurobasal [Gibco], N2 [Gibco] 1:100, B27 [Gibco] 1:50, Glutamax [Gibco] 1:100, Non-essential Amino Acids [Gibco] 1:100, β-mercaptoethanol [Gibco] 1:1000) containing 20 ng/mL FGF-2 (R&D Systems), 5 μM Y-27632 (Generon), 0.2 μM LDN193189 (Tocris), 4 μM Chir99021 (Tocris), 40 μM SB431542 (Tocris), and 0.05% poly(vinyl alcohol) (Sigma-Aldrich). Plates were spun at 1400 rpm for 4 minutes, and 50% media changes were performed every 2 days unless otherwise stated. On day 2, medium was changed to contain 20 ng/mL FGF-2, 0.2 μM LDN193189, 4 μM Chir99021, 40 μM SB431542, 1 μM all-*trans* retinoic acid (Sigma-Aldrich), and 0.5 μM SAG (Tocris). On day 4, medium contained 1 μM all-*trans* retinoic acid and 0.5 μM SAG. On day 7, medium contained 1 μM all-*trans* retinoic acid, 0.5 μM SAG, 10 ng/mL BDNF (Peprotech), and 10 ng/mL GDNF (Peprotech). On day 9, medium was changed to contain 10 μM DAPT (Tocris), 10 ng/mL BDNF, 10 ng/mL GDNF, 1 μM all-*trans* retinoic acid, and 0.5 μM SAG. On day 13, spheres were pooled together, dissociated using Aaccutase, and replated on poly-L-ornithine (Sigma-Aldrich, P4957) and GelTrex-coated dishes in media containing 10 μM DAPT, 10 ng/mL BDNF, 10 ng/mL GDNF, 0.1 μM retinoic acid, and 0.5 μM SAG. On day 14, retinoic acid and SAG were removed from the culture media and DAPT was increased to 20 μM. ON day 16, 10 ng/mL CNTF (Peprotech) was added to the culture media. On day 17, DAPT was removed from the media. From this point on, media contained only BDNF, GDNF, and CNTF at 10 ng/mL. Cells were used for experiments on day 16 or 34 as stated.

hiPSCs were differentiated as previously described in Van Lent et al. (33) on microfluidic devices (Supplemental Figure 6C).

### Differentiation of hESCs into enteric neurons.

Enteric neuron differentiation was carried out using a modified version of a previously published protocol (62). Briefly, hESCs were seeded at 40,000 cells/cm^2^ on vitronectin-coated (Thermo Fisher Scientific) plates in vagal neural crest medium (DMEM-F12 [Gibco], 1:100 GlutaMAX [Gibco], 1:100 MEM-NEAA [Gibco], 1:100 N2-B [Stem Cell Technologies], 1 μM CHIR99021 [Tocris], 10 μM Y-27632 [Generon], 20 ng/mL BMP4 [Thermo Fisher Scientific], 1 μM SB421542 [Tocris], and 200 nM LDN193189 [Tocris]). Y-27632 was removed from the media on day 2. All-*trans* retinoic acid was added to the media on day 4 and day 5 at a final concentration of 1 μM. On day 6, the resulting vagal neural crest cells were replated at 150,000 cells/cm^2^ on GelTrex-coated Ibidi dishes in enteric nervous system (ENS) induction medium (Neurobasal [Gibco], 1:100 N2-B, 1:100 GlutaMAX, 1:50 B27 [Gibco], 100 μM ascorbic acid [Sigma-Aldrich], 25 ng/ml GDNF [Peprotech], and 10 μM Y-27632). On day 7, replated cells were fed with ENS induction medium without Y-27632. Cells were fed every day for a further 12 days by 50% media changes.

### Time-lapse analysis of mitochondrial movement.

Motor neurons (differentiation day 13) were seeded into PLO/GelTrex-coated 35 mm dishes (Ibidi, 81156) and differentiated as detailed above. On day 34 of differentiation, neurons were transfected with a 1:1 ratio of pCAG GFP (63) and DsRed2-Mito (Tanaka Bio) plasmids using Lipofectamine LTX Plus (Thermo Fisher Scientific, 15338100) for 6 hours, before fresh media replacement. A total of 1 μg of plasmid DNA was added to each 35 mm Ibidi dish. Treatment, if applicable, was added 24 hours after transfection. Neurons were imaged 48 hours after transfection in widefield using a custom imaging system (Cairn Research) at 37°C. This system uses a Ti2-E automated inverted microscope base (Nikon), ASI automated XY and piezo-Z stage controller, iLas 2 scanning TIRF/FRAP unit (Gataca Systems) with dual-collimation optics, Cairn MultiLine LaserBank, and a Photometrics Prime 95B sCMOS camera. Imaging was performed with a 488 nm and 561 nm laser, a quad-band filter cube (TRF89901-EMv2, Chroma), and additional emission clean up provided by a Cairn Optospin filter wheel with ET525/50m or ET595/44m filters for 488 and 561 nm laser lines, respectively. Images were acquired in MetaMorph acquisition software using a Nikon CFI Apochromat Lamda 60× oil (N.A. 1.40, W.D. 0.13 mm) objective lens. For time-lapse imaging, images of DsRed2 were taken every 500 milliseconds for 2.5 minutes, and GFP images were taken in the first and last frames. Recordings were made in the proximal regions (within 500 μm from cell body), mid regions (500 μm to 1500 μm from the cell body), and distal regions (>1500 μm from the cell body) of the neuron. Two to 4 neurons per cell line per condition were recorded from each independent differentiation. The first frame of each time lapse was thresholded and the area and shape descriptors were calculated for mitochondria within the axon of interest. Kymographs were generated from time-lapse images using a custom-made FIJI macro. Kymographs were segmented and run through KymoButler (64) to generate track predictions. Kymograph segments with KymoButler track predictions were restitched in R, and motility and velocity measures were calculated from here (Supplemental Figure 8). After each time lapse, the entire neuron was imaged using GFP to calculate the total length and where each individual recording was in relation to the cell body. Mitochondrial morphology in the axon was calculated by taking the first frame of each kymograph and “Analyze Particles” was run on a thresholded image to measure individual mitochondria. Instantaneous velocity was calculated by manually adding short (<40 pixels) straight lines to individual discrete movements on kymographs and calculating the distance covered over change in time. Whole-track velocity was calculated by taking the total distance covered over change in time of a single motile track. The pause coefficient was determined by calculating how long a track spent paused via a sliding window of 4 seconds and summing this value for all motile tracks in a kymograph, then dividing this by the total distance travelled by all motile tracks in a kymograph to normalize. Velocity variation was calculated using a sliding window of 4 seconds to calculate velocity across a motile track.

### Protein extraction.

Adherent cells were washed in phosphate-buffered saline (PBS; 137 mM NaCl, 2.7 mM KCl, 1.5 mM KH_2_PO_4_, 10 mM Na_2_PO_4_•2H_2_O), scraped into Laemmli buffer (60 mM Tris HCl pH 6.8, 2% SDS, 0.002% bromophenol blue, 5% β-mercaptoethanol, 5% glycerol) and 1× DNase I (Thermo Fisher Scientific). Protein samples were denatured at 100°C for 5 minutes before analysis by immunoblotting.

### Endogenous IP.

hESCs were washed with cold PBS and harvested into modified Tris buffer (50 mM Tris pH 7.5, 150 nM NaCl, 2 mM EDTA, 5% w/v glycerol, 1% w/v NP-40, 1× Halt Protease and Phosphatase Inhibitor Cocktail [Thermo Fisher Scientific]) and lysed at 4°C for 30 minutes. Lysates were cleared by centrifugation at 15,000*g* for 20 minutes at 4°C, and 6 mg protein was incubated with 5 μL α-MFN2 primary antibody (Cell Signaling Technology) overnight at 4°C. Forty microliters of Protein A Mag Sepharose beads (GE Healthcare) was added to the samples and incubated for 3 hours at 4°C. Beads were washed with buffer and eluted for downstream experiments with 30 μL 5% SDS/50 mM Tris, pH 7.4 and heating at 70°C for 15 minutes.

### MS analysis of MFN2 IPs.

Twenty-five microliters of IP elution (5% SDS/50 mM Tris, pH 7.4) was reduced with TCEP added to a final concentration of 5 mM and heated at 70°C for 15 minutes. Proteins were alkylated by adding iodoacetamide (IAA) to a final concentration of 10 mM, and the samples were incubated at 37°C in the dark for 30 minutes. Two and one-half microliters of 12% phosphoric acid and 165 μL of S-Trap binding buffer (90% methanol, 100 mM TEAB, pH 7.1) were then added to each sample. Samples were then loaded into S-Trap columns (ProtiFi) by centrifugation at 10,000*g* for 60 seconds. Samples were washed 4 times with 225 μL of S-Trap binding buffer through centrifugation at 10,000*g* for 60 seconds. One microgram of trypsin (Pierce, sequencing grade) was added to each sample in a total digestion volume of 20 μL of 50 mM TEAB, and digestion was performed at 47°C for 1 hour, followed by 37°C for 1 hour. Peptides were eluted with the addition of 40 μL of 50 mM TEAB, 40 μL of 0.2% aqueous formic acid, and then 40 μL of 50% acetonitrile with 0.2% formic acid, followed by centrifugation at 10,000*g* for 60 seconds for each elution. Pooled eluted peptides were dried in a vacuum concentrator and resuspended in 0.5% formic acid for LC-MS/MS analysis. Each sample was analyzed using nanoflow LC-MS/MS using an Orbitrap Exploris 480 (Thermo Fisher Scientific) mass spectrometer equipped with an EASY-Spray source coupled to a Vanquish LC System (Thermo Fisher Scientific). Peptides were desalted online using a Pepmap Neo C18 nano trap column (300 μm ID × 5 mm; Thermo Fisher Scientific) and then separated using an EASY-Spray column (50 cm × 75 μm ID, PepMap Neo C18, 2 μm particles, 10 Å pore size; Thermo Fisher Scientific) and separated using a 70-minute gradient. The Orbitrap Exploris 480 was operated in positive mode using a Velocity DIA method. MS1 spectra were acquired at a resolution of 60,000 at *m*/*z* 200 with a normalized AGC target of 300%. For the DIA analysis, multiply charged precursors in 40 *m*/*z* bins (400–900 *m*/*z* range) were accumulated with a normalized AGC target of 800%, subjected to HCD fragmentation (145–1500 *m*/*z*) with a collision energy of 30%.

Raw MS data files were processed with DIA-NN version 2.3.0 (https://github.com/vdemichev/diann). Data were analyzed in library-free mode using a peptide library predicted from a Human proteome Fasta file (downloaded Aug 2025) (https://www.uniprot.org/proteomes/UP000005640) containing 20,475 proteins. The following settings were used in library prediction. Trypsin was set as the protease, with a maximum of 1 missed cleavage. Cysteine carbamidomethylation was enabled as a fixed modification. Maximum number of variable modifications set to 1 and acetyl-protein N term set as a variable modification. An FDR of 1% used for identification-level cutoffs. The DIA-NN output was loaded into Perseus version 1.6.10.50 (https://maxquant.net/perseus/), and the matrix was filtered to remove all proteins that were potential contaminants and decoy hits. Label-free quantification (LFQ) intensities were log_2_(*x*) transformed, and data were filtered to retain proteins with a minimum of 2 peptides and at least 3 valid LFQ intensities in one group. Data were normalized by subtracting column medians, and missing values were imputed from the normal distribution with a width of 0.3 and downshift of 1.8. To identify quantitatively enriched proteins in MFN2 IPs versus control IPs, 2-sided Student’s *t* tests were performed with a permutation-based FDR of 0.05. To identify differences in MFN2 interactions between variant and wild type, only proteins significantly enriched in IP versus controls were considered. LFQ intensities were instead normalized to the bait protein, and missing values were imputed from the normal distribution with a width of 0.3 and downshift of 1.8, and 2-sided Student’s *t* tests were performed with a permutation-based FDR of 0.05.

### Western blotting.

Proteins were separated by SDS-PAGE and transferred to nitrocellulose membranes (Whatman, Thermo Fisher Scientific) by electroblotting (Bio-Rad). After transfer, membranes were blocked for 1 hour at room temperature in Tris-buffered saline (TBS) with 5% fat-free milk and 0.1% Tween 20. Membranes were incubated with primary antibodies in blocking buffer overnight at 4°C. Membranes were washed 3 times for 5 minutes in TBS with 0.1% Tween 20 before incubation with near-infrared Alexa Fluor–coupled secondary antibodies in TBS with 0.1% Tween 20 for 1 hour at room temperature. Membranes were washed 3 times for 5 minutes in TBS with 0.1% Tween 20 before near-infrared fluorescent signals were detected using an Odyssey XF Imaging System with ImageStudio software (LI-COR Biosciences). For primary and secondary antibodies used in immunoblotting, see Supplemental Tables 6 and 7.

### Electrophysiology.

Whole-cell patch clamp was used to record membrane currents or membrane potentials from single cells (*n* = 33) at room temperature (20°C–25°C) using an Optopatch (Cairn Research Ltd) patch clamp amplifier. The extracellular solution contained (mM): 135 NaCl, 5.8 KCl, 1.3 CaCl_2_, 0.9 MgCl_2_, 0.7 NaH_2_PO_4_, 5.6 D-glucose, 10 HEPES-NaOH, 2 sodium pyruvate. Amino acids and vitamins for Eagle’s minimal essential medium (MEM) were added from concentrates (Invitrogen). The pH was adjusted to 7.5 and the osmolality was approximately 308 mOsmol/kg. Cells were viewed using an upright microscope equipped with Nomarski DIC optics (Nikon FN1) and were continuously perfused with extracellular solution. Patch electrodes were pulled from soda glass capillaries (Hilgenberg GmbH) and electrodes had resistances in extracellular solution of approximately 4 MΩ. The shank of the electrode was coated with surf wax (Mr Zogs Sexwax) to minimize the fast electrode capacitive transients. The pipette solution contained (mM): 131 KCl, 3 MgCl_2_, 1 EGTA-KOH, 5 Na_2_ATP, 5 HEPES-KOH, 10 sodium phosphocreatine (pH 7.3, 290 mOsmol/kg). Voltage and current clamp protocol application and data acquisition were performed using pClamp software and a Digidata 1440A (Molecular Devices). Recordings were filtered at 2.5 or 10 kHz (8-pole Bessel), sampled at 5 or 100 kHz, and stored on a computer for off-line analysis using Clampfit, GraphPad Prism (GraphPad Software Inc), and Origin (OriginLab) software. Recordings and reported currents and conductances were corrected off-line for the linear leak conductance. Membrane potentials under voltage clamp were corrected for the voltage drop across the residual series resistance (*R*_s_) at steady-state current level and for a liquid junction potential, measured between pipette and bath solutions, of –4 mV.

### Statistics.

Statistical analysis was performed using GraphPad Prism version 9.3.1 or R by statistical tests as indicated in figure legends. Data were tested for normality via a Shapiro-Wilks test and distribution observations, and descriptive statistics were determined to examine the SD for each sample. If data were found to be normally distributed, then a 2-tailed Student’s *t* test or ANOVA with Dunnett’s T3 multiple-comparison test was used. If data were found to not be normally distributed, a Mann-Whitney *U* test or a Kruskal-Wallis test with Dunn’s multiple-comparison test was performed. For all the statistical tests, a *P* value of less than 0.05 was used as the criterion for statistical significance.

### Study approval.

Zebrafish experiments were conducted according to UK law under project license 40/8309 granted to AJG, and were subject to a local ethical review process.

### Data availability.

Code that was used to analyze Kymobutler outputs is deposited in ORDA (https://doi.org/10.15131/shef.data.30196639.v1), the University of Sheffield data repository, powered by Figshare.

The MS proteomics data have been deposited to the ProteomeXchange Consortium via the PRIDE partner repository with the dataset identifier PXD077751.

Values for all data points in graphs are reported in the Supporting Data Values file.

## Author contributions

LHJ, LB, RAL, KIA, JVL, SLJ, HW, BA, EB, OL, CJP, ED, MOC, and DS conducted experiments and acquired data. KJDV and IB provided reagents. LHJ, LB, KIA, JVL, SLJ, BA, AT, MOC, VT, KJDV, AET, AJG, and IB designed research studies and analyzed data. LHJ, LB, AJG, and IB wrote and edited the manuscript. JVL, BA, OL, GG, AT, VT, KJDV, AET, and AJG edited the manuscript.

## Conflict of interest

The authors have declared that no conflict of interest exists.

## Funding support

- Muscular Dystrophy UK grant 22GRO-PG24-0571-1 (to IB and AJG).
- Medical Research Council (MRC) grants MR/X000028/1, MR/X007979/1, and MR/X012220/1.
- Hereditary Neuropathy Foundation (to IB).
- MRC DiMeN studentship MR/W006944/1.
- Wellcome Trust and the Royal Society Sir Henry Dale Fellowship 220192/Z/20/Z (to AET).
- University of Antwerp DOC-PRO4 PhD fellowship (to JVL), TOP-BOF research grant N_38694 (to VT).
- VT is a member of the μNeuro Center of Excellence at the University of Antwerp.
- MRC grant MR/S025979/1 (to KJDV).
- Alzheimer’s Society grant AS-PG-15-023 (to KJDV).
- Alzheimer’s Research UK project grant ARUK-PG2019A-008 (to KJDV).

## Supplementary Material

Supplemental data

Unedited blot and gel images

Supplemental video 1

Supplemental video 2

Supporting data values

## Version 1 07/14/2026

In-Press Preview

## Version 2 08/24/2026

Electronic publication
