# Supplementary material for: HDAC6 inhibition alleviates mitochondrial trafficking in models of Charcot-Marie-Tooth disease type 2A
Source: JCI Insight. 2026 Jul 14;11(16):e200106. doi: 10.1172/jci.insight.200106 (PMC13502185; doi:10.1172/jci.insight.200106)
Supplement: Supplementary file 1 [file jciinsight-11-200106-s131.pdf]

Supplementary Figures

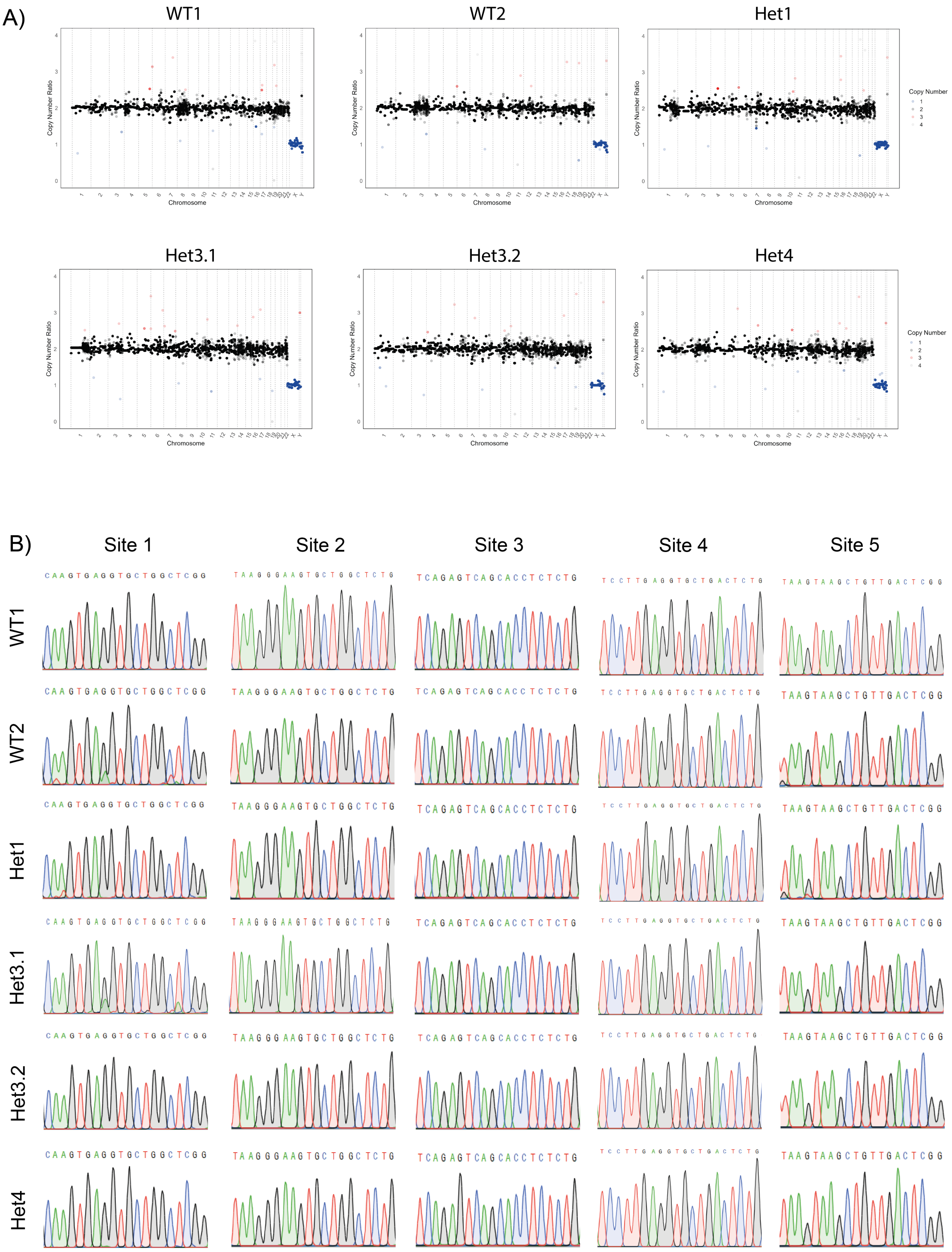

Supplementary Figure 1: Genetic analysis of wild-type and variant MFN2 hESC clones. (**A**) Manhattan plots of chromosomal copy number in clones that underwent genetic editing. Unedited MShef11 hESCs (*MFN2*$^{+/+}$ WT1) were included as a control. (**B**) Sanger sequencing for the top 5 most likely sites for off-target editing revealed no off-target variants in clones that underwent genetic editing. Unedited MShef11 hESCs (*MFN2*$^{+/+}$ WT1) were included as a control.

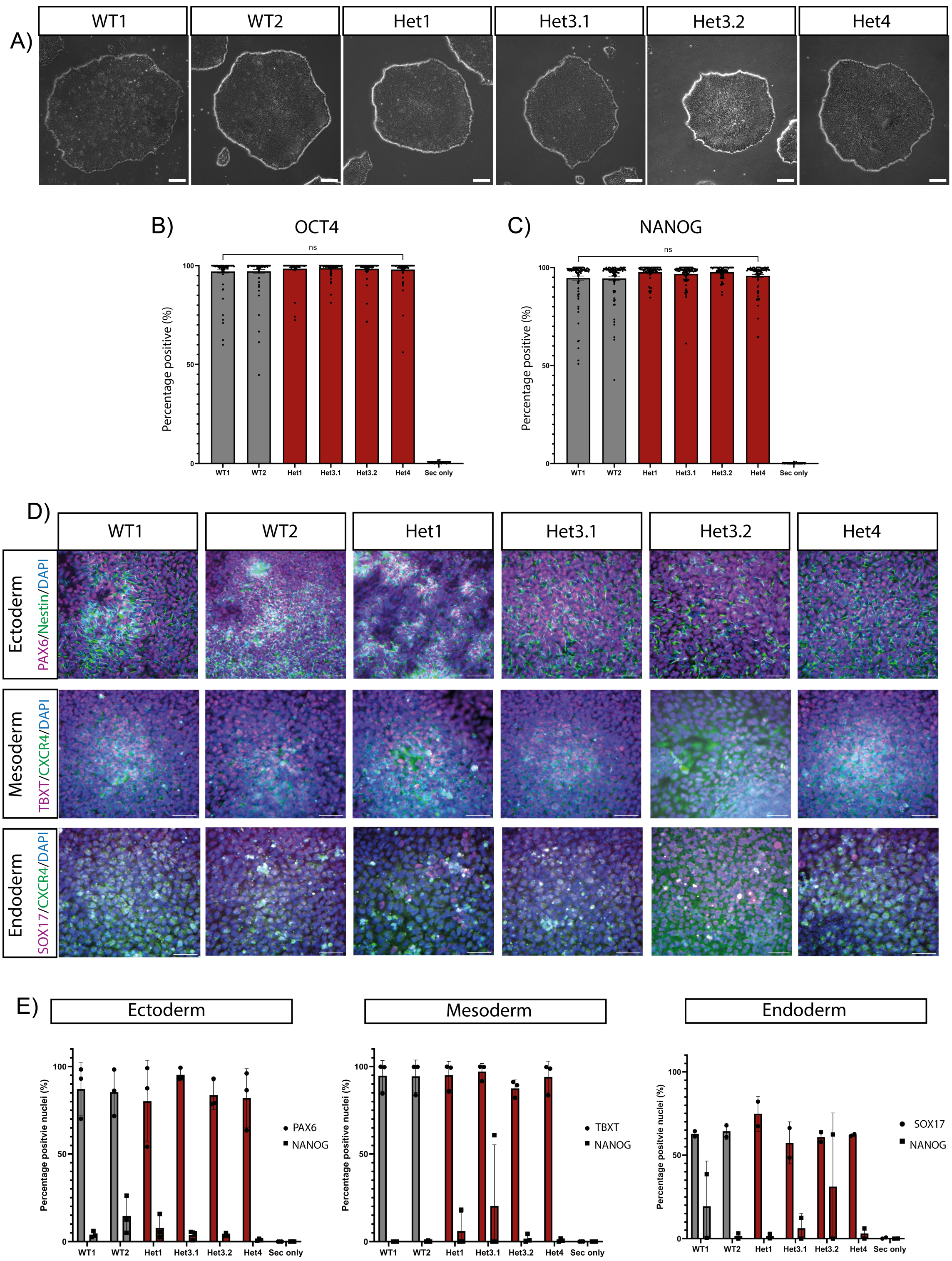

Supplementary Figure 2: Assessment of pluripotency of wild-type and variant MFN2 hESC clones. (**A**) Representative phase contrast images of *MFN2*$^{R94Q/+}$ and *MFN2*$^{+/+}$ hESC clones. Scale bar = 100 µm. (**B**) Percentage of nuclei positive for the pluripotency-associated markers OCT4 and (**C**) NANOG. Data shown at mean ± SEM, N = 3 independent biological repeats. (**D**) Representative immunofluorescence images of *MFN2*$^{R94Q/+}$ and *MFN2*$^{+/+}$ hESC lines subjected to endodermal, ectodermal, or mesodermal differentiation using the StemCell Technologies STEMdiff Trilineage differentiation kit. Scale bar = 200 µm. (**E**) Quantification of percentage positive nuclei from (D). N = 2-3 independent differentiations.

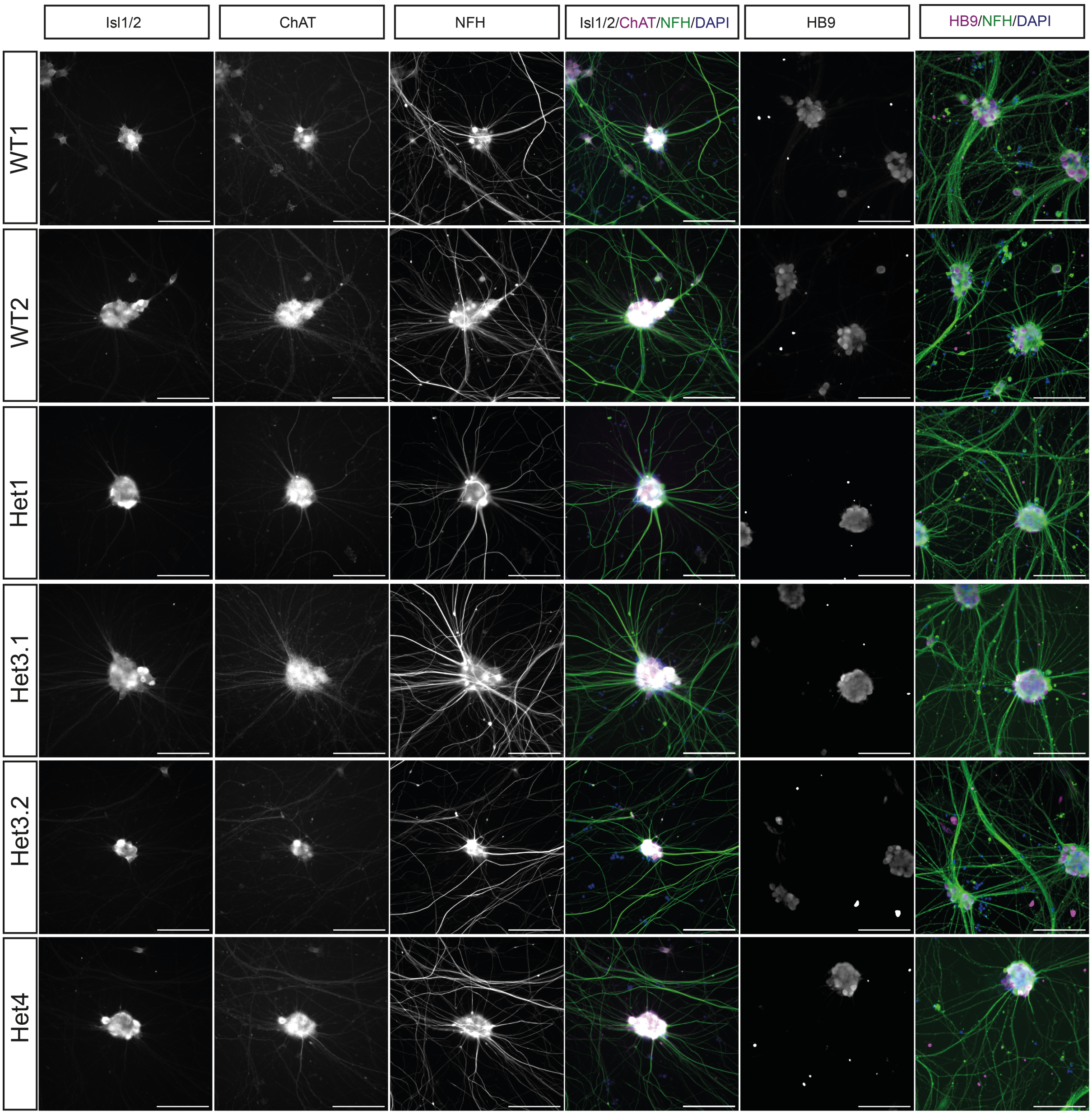

Supplementary Figure 3: Visualisation of motor neuron differentiation in all wild-type and variant MFN2 hESC clones by immunofluorescence. Representative immunofluorescence images of *MFN2*$^{R94Q/+}$ and *MFN2*$^{+/+}$ hESC-derived motor neurons immunoreactive to key motor neuron markers. Scale bar = 100 µm.

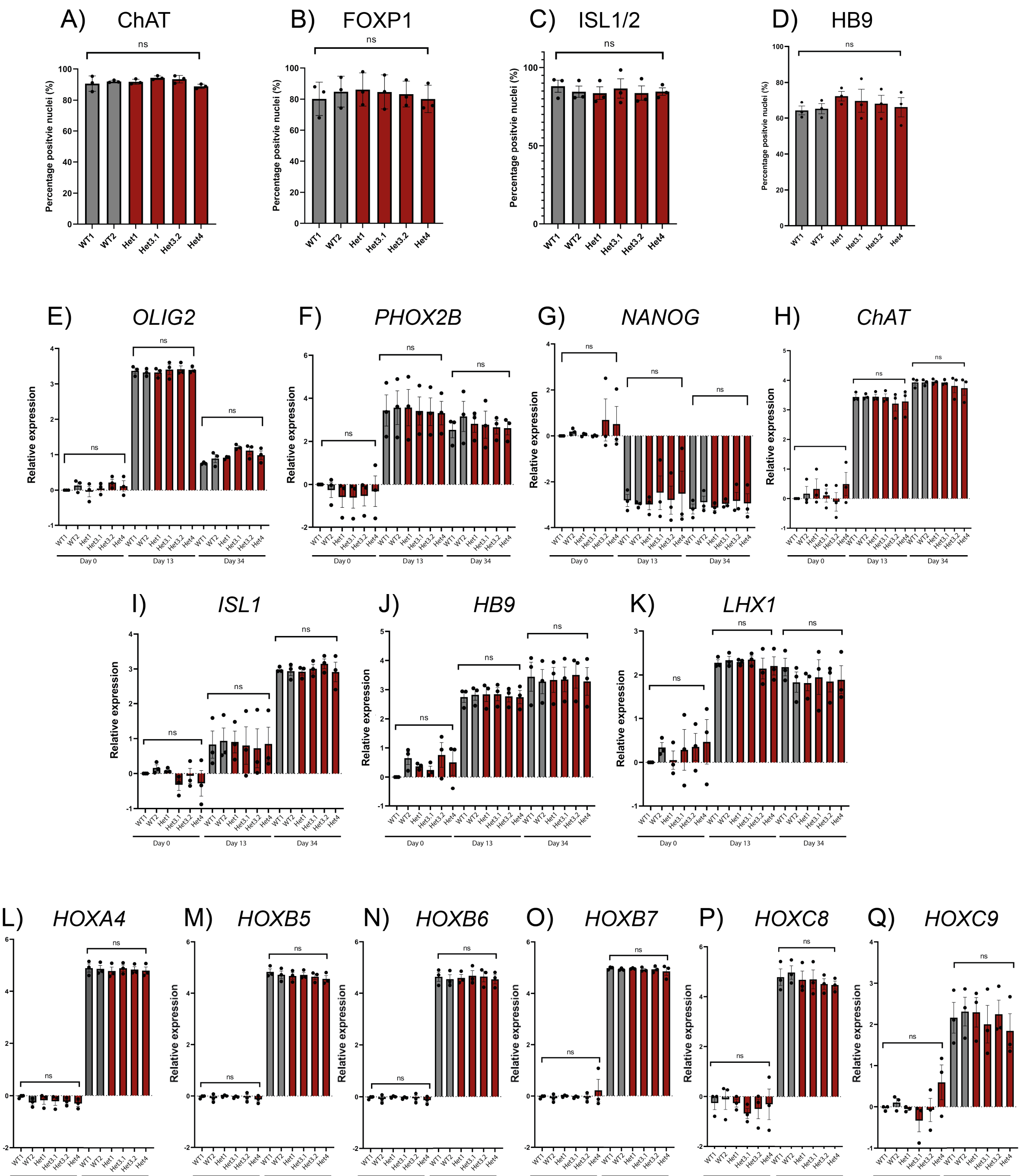

Supplementary Figure 4: Assessment of motor neuron differentiation in all wild-type and variant MFN2 hESC clones. (**A-D**) Quantification of percentage positive nuclei immunoreactive for motor neuron associated markers at Day 34 of differentiation. Data shown as mean ± SEM, N = 3 independent biological repeats. (**E-K**) RT-qPCR analysis of motor neuron-associated markers in wild-type and variant lines at Day 0, Day 13, and Day 34 of differentiation, normalised to WT1 Day 0. (**L-Q**) RT-qPCR analysis of HOX gene expression in wild-type and variant lines at Day 0 and Day 34 of differentiation, normalised to WT1 Day 0. Data shown as mean ± SEM, N = 3 independent biological repeats, Kruskal-Wallis test, ns = p>0.05.

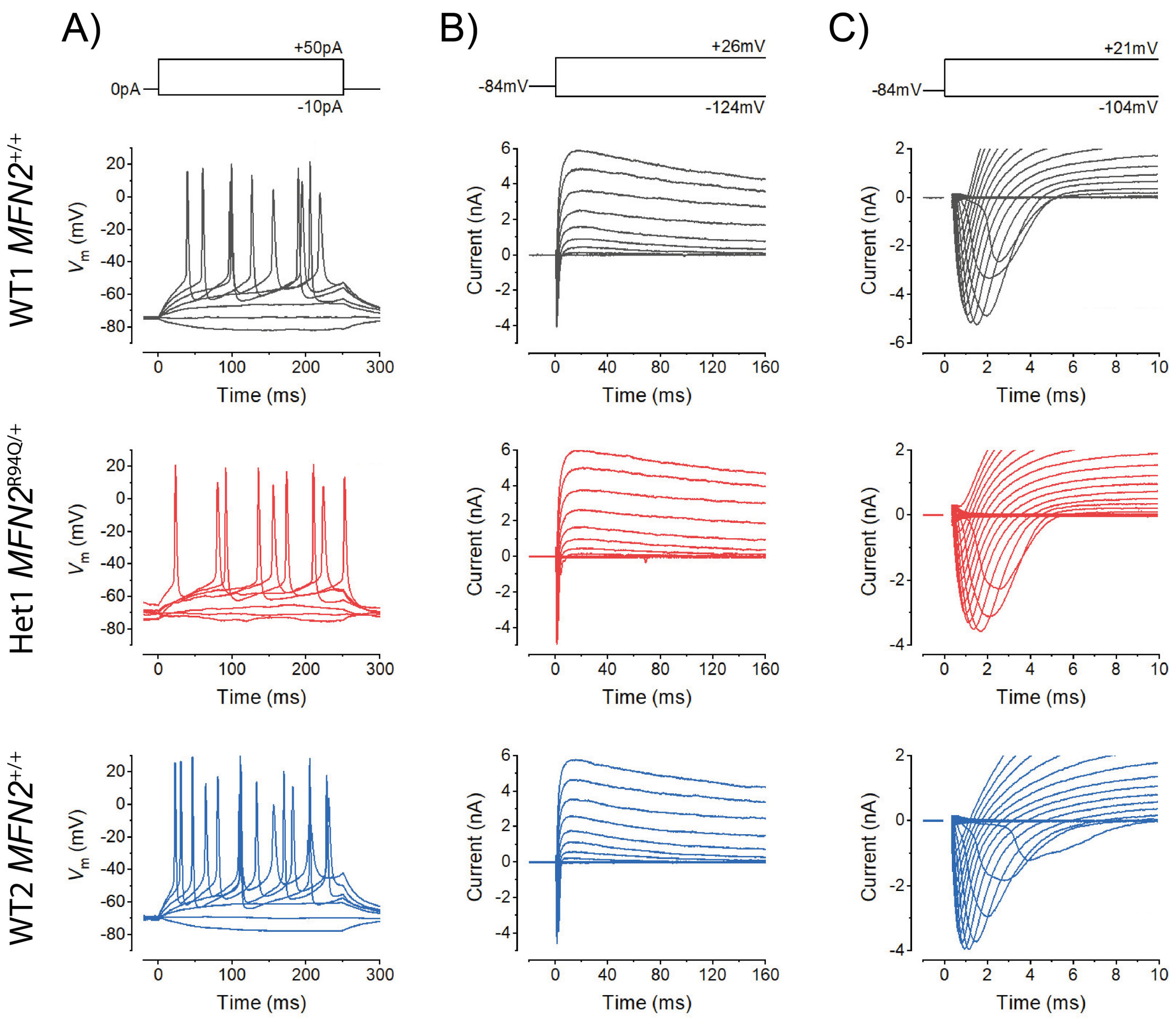

Supplementary Figure 5: Electrophysiology of hESC-derived spinal motor neurons. (**A**) Membrane potential responses from WT1 (black, upper panel) and Het1 (red, middle panel) and WT2 (blue, lower panel) neurons in response to depolarising current injection. Cells were stimulated with the current clamp protocol shown above. (**B**) Inward Na^+ (I_{Na}, negative) and outward K^+ (I_K, positive) currents from WT1 (black, upper panel) and Het1 (red, middle panel) and WT2 (blue, lower panel) neurons recorded in voltage clamp in response to the voltage protocol shown above. (**C**) Inward I_{Na} from WT1 (black, upper panel) and Het1 (red, middle panel) and WT2 (blue, lower panel) neurons in response to 10 ms voltage steps in 5 mV increments (voltage protocol shown above).

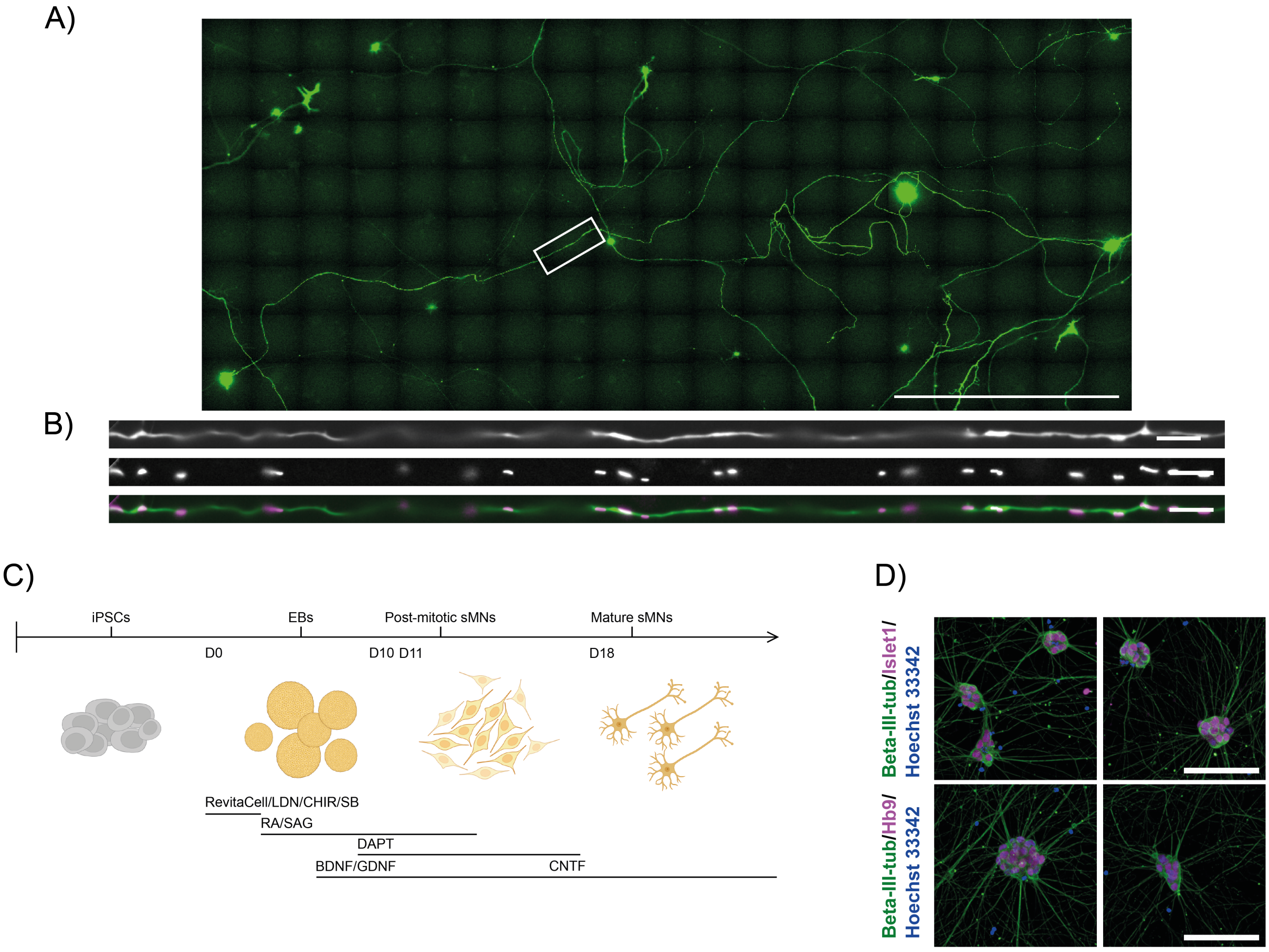

Supplementary Figure 6: Neuronal models for observing mitochondria. (**A**) Low efficiency transfection of dense neuronal cultures isolates individual neurons. Scale bar = 1000µm. (**B**) Magnified section of (A) shows dsRed-positive mitochondria within a GFP-positive neuron. Scale bar = 10 µm. (**C**) Differentiation schematic for generating spinal motor neurons (sMNs) from human iPSCs as previously used in Van Lent et al., 2021. (**D**) Motor neuron marker analysis by immunofluorescence in spinal motor neurons derived from (C), as previously shown in Van Lent et al., 2021.

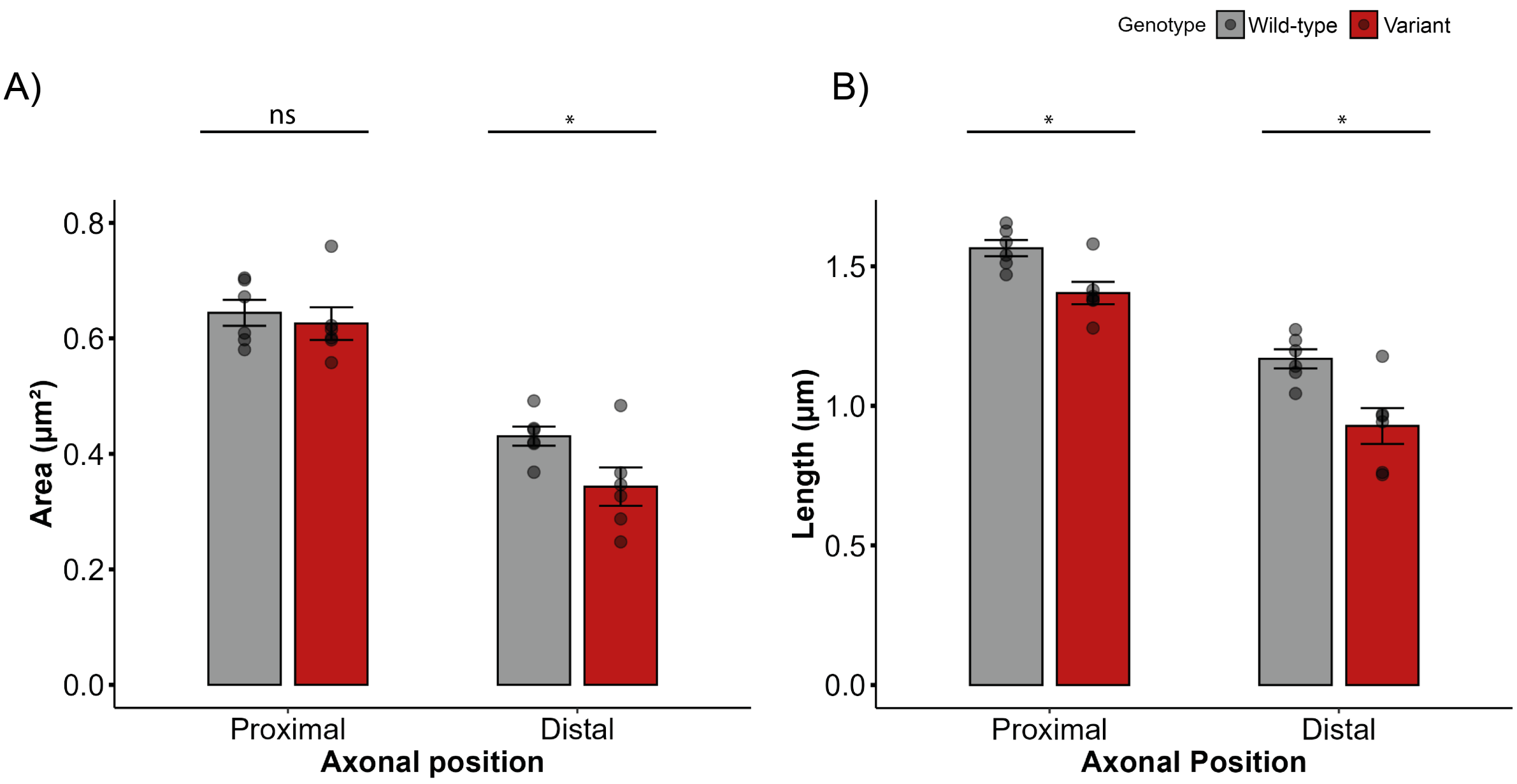

Supplementary Figure 7: Mitochondrial size in iPSC-derived motor neurons. (**A**) Average area of mitochondria in wild-type and variant *MFN2*$^{R94Q/+}$ iPSC-derived motor neurons. (**B**) Average length of mitochondria in wild-type and variant *MFN2*$^{R94Q/+}$ iPSC-derived motor neurons. Data shown as mean ± SEM with points representing average values from individual fields of view. Statistical significance was calculated with a Wilcoxon rank sum test (N = 6 wild-type, 12 variant) (*P<0.05; **P<0.01; ***P<0.001; ns, not significant).

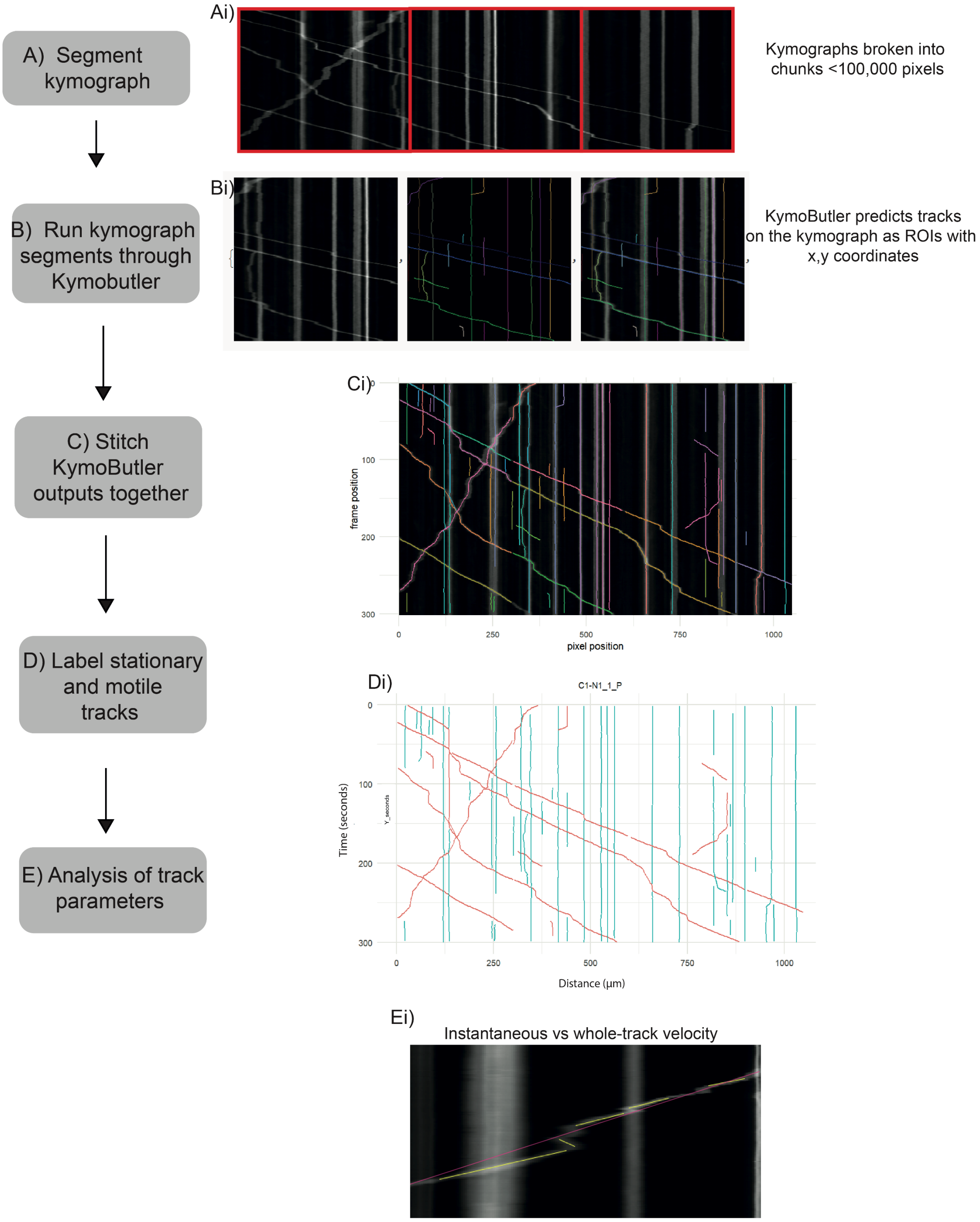

Supplementary Figure 8: Kymograph analysis workflow. Kymographs were segmented (**A, Ai**) into chunks <100,00 pixels due to Kymobutler limitations and run through Kymobutler with a detection threshold of 0.15, minimum particle size of 15 and minimum frame number of 30 (**B, Bi**). Kymobutler track coordinates and corresponding kymograph chunk were restitched together to re-form the kymograph using R (**C, Ci**). Tracks were filtered into motile or stationary (**D, Di**), and end-joining was performed to join gaps in tracks left by the initial segmenting of the kymograph. The analysis of track parameters was performed on these tracks (**E**). A sliding windows approach calculated velocity across a motile track. Whole track velocity is shown by the pink line and instantaneous velocity by the yellow lines (**Ei**).

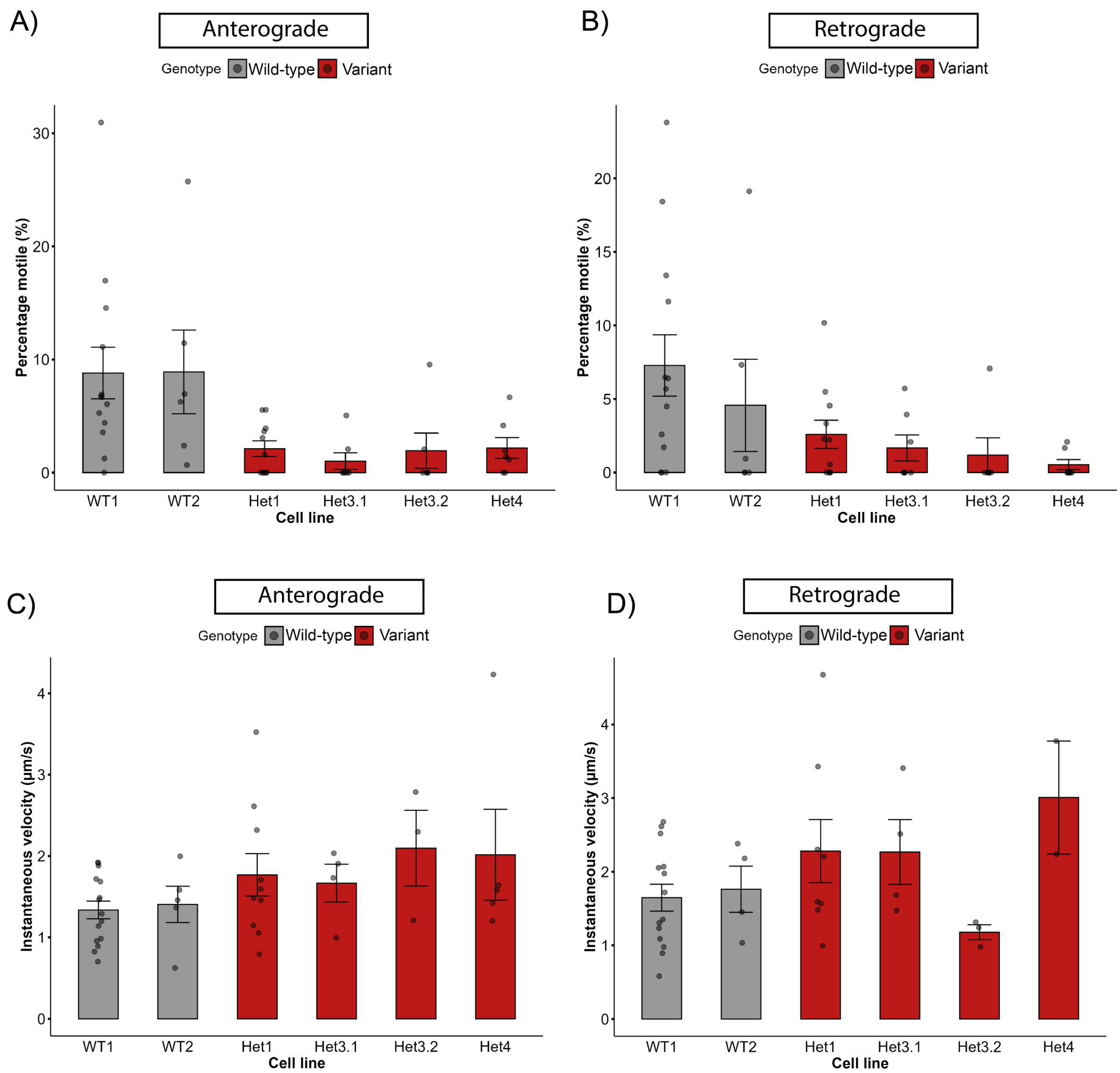

Supplementary Figure 9: Mitochondrial trafficking deficit. (**A**) Anterograde trafficking deficit and retrograde (**B**) trafficking deficit is seen in all variant clones. (**C**) Anterograde and retrograde (**D**) instantaneous velocity in all lines. Data points represent values for individual neurons, plotted as mean ± SEM.

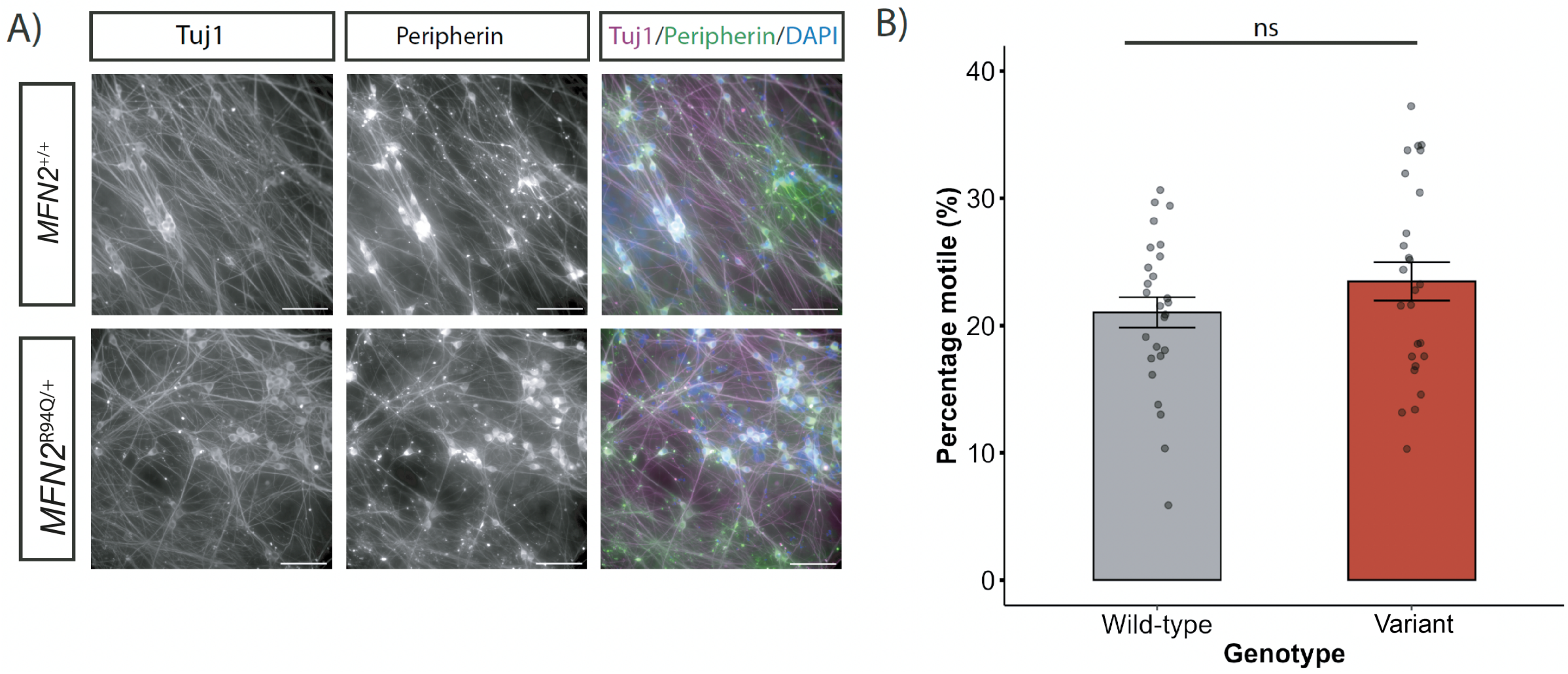

Supplementary Figure 10: Mitochondrial trafficking deficit is not present in enteric neurons. (**A**) Representative immunofluorescence images of *MFN2*$^{+/+}$ and *MFN2*$^{R94Q/+}$ hESC-derived enteric neurons. Scale bar = 100 µm. (**B**) The percentage of motile mitochondria in hESC-derived enteric neurons. Data shown as mean ± SEM with points representing average values from individual neurons. Statistical significance was calculated with a Wilcoxon rank sum test (N = 4 wild-type, 4 variant) (ns, not significant).

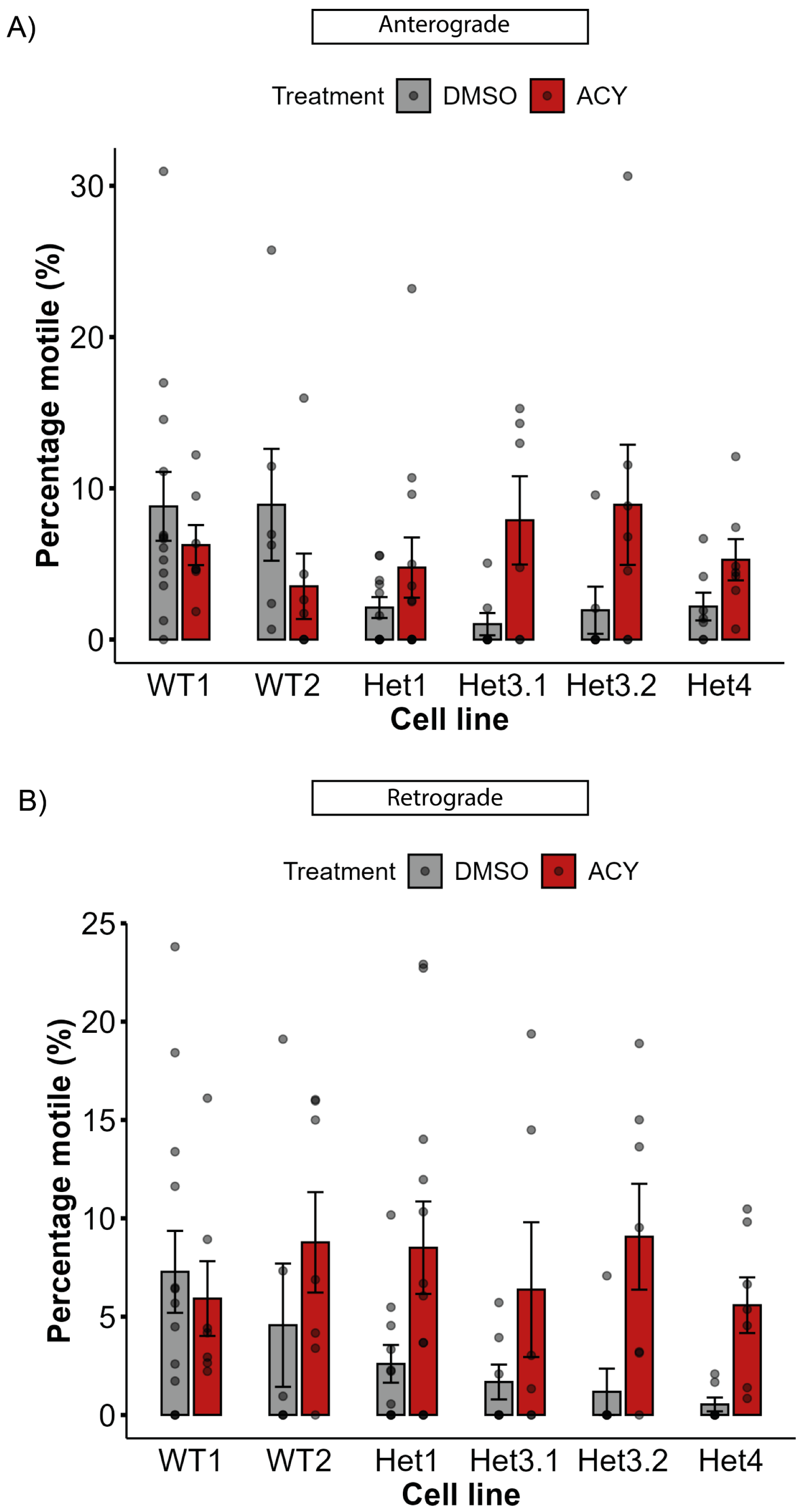

Supplementary Figure 11: ACY-738 treatment increases anterograde and retrograde mitochondrial movement. (**A**) Anterograde trafficking deficit and retrograde (**B**) trafficking deficit is rescued in all variant clones. Data points represent values for individual kymographs plotted as mean ± SEM, from 3 independent differentiations.

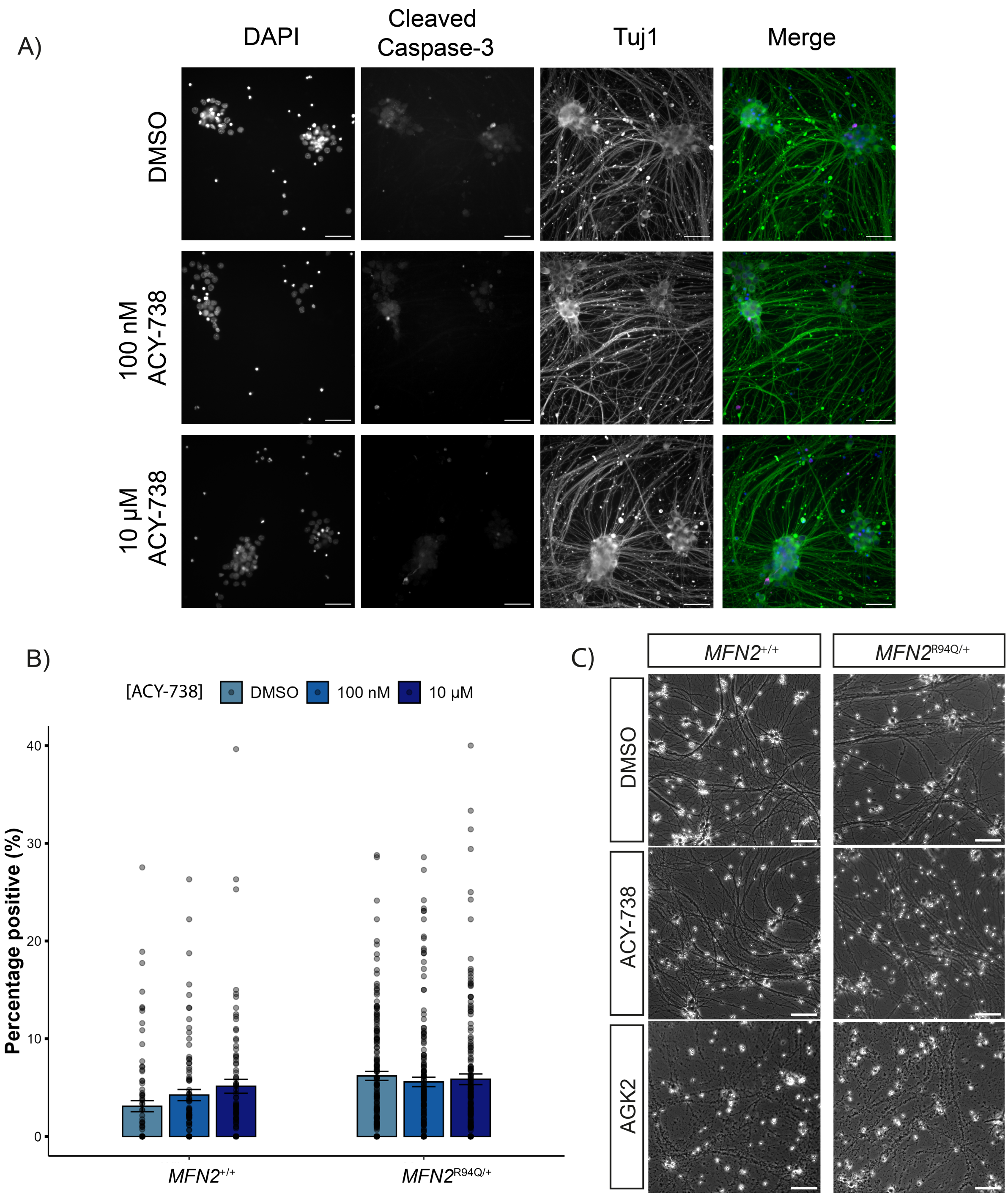

Supplementary Figure 12: ACY-738 does not exert overt toxicity in hESC-derived motor neurons after 24-hour treatment. (**A**) Representative immunofluorescence images of Het1 after 24-hour treatment with 0.1 % DMSO, 100 nM ACY-738, or 10 µM ACY-738. Scale bar = 50 µm. (**B**) Quantification of the percentage of nuclei positive for cleaved caspase-3 after 24-hours of DMSO or ACY-738 treatment. Data points represent individual fields of view plotted as mean ± SEM, N = 1 independent differentiation. (**C**) Representative phase contrast images of hESC-derived neurons after 24-hour treatment with DMSO, 100 nM ACY-738, or 5 µM AGK2. Scale bar = 200 µm.

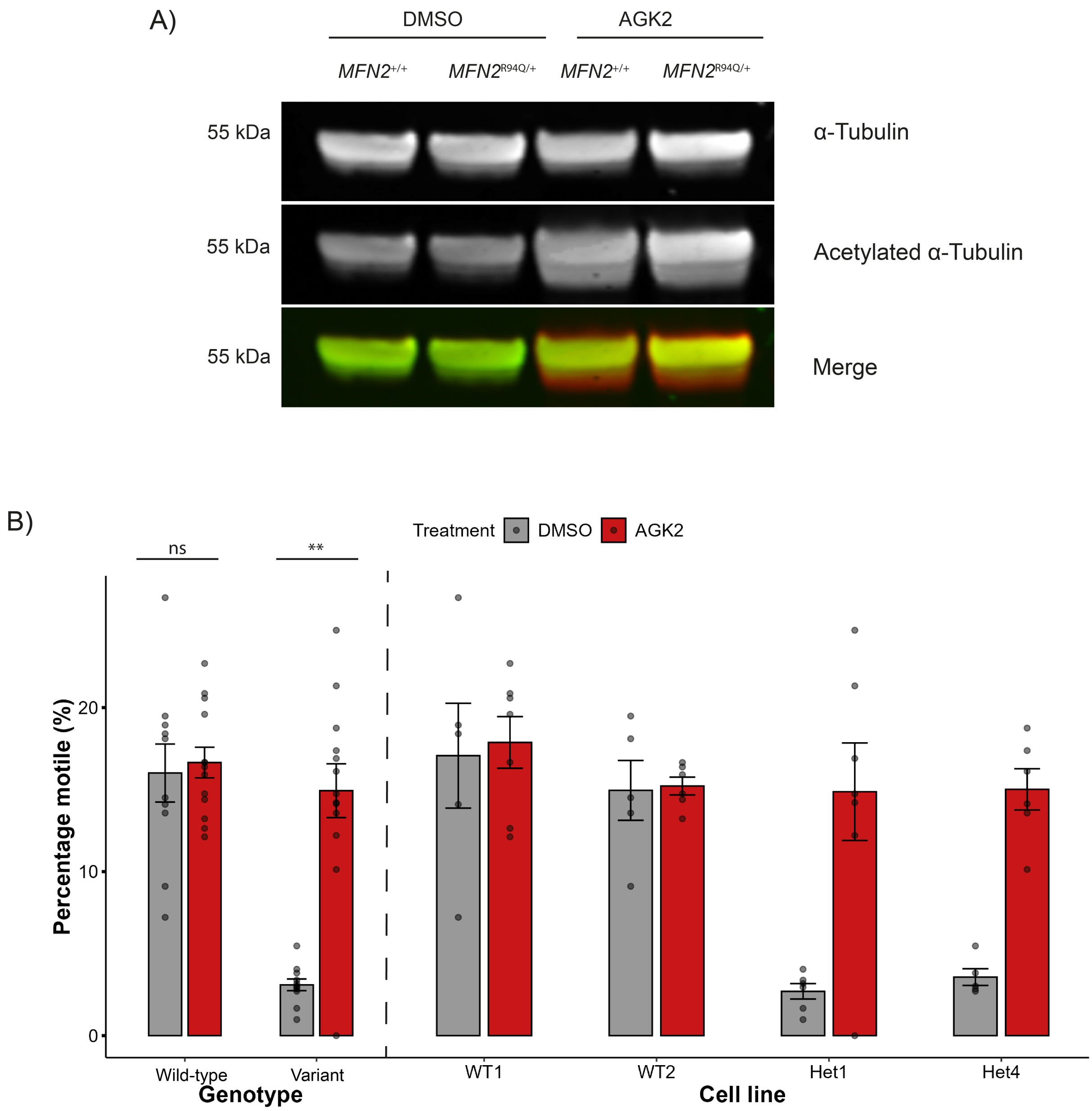

Supplementary Figure 13: SIRT2 inhibition increase mitochondrial transport in MFN2$^{R94Q/+}$ hESC-derived motor neurons. (**A**) Western blot analysis of acetylated tubulin levels in hESC-derived motor neurons treated for 4 hours with 0.05 % DMSO or 5 µM of the SIRT2 inhibitor AGK2. (**B**) Quantification of the percentage of motile mitochondria in DMSO- or AGK2-treated control and variant lines. Data shown as mean ± SEM with points representing average values from individual neurons. Statistical significance was calculated with a Wilcoxon rank sum test (**, P< 0.01). N = 3 independent differentiations.

Supplementary Movies

Supplementary Movie 1. Representative time-lapse video of dsRed-labelled mitochondria in axons of wild-type *MFN2*$^{+/+}$ hESC-derived spinal motor neurons. One image was taken every 500ms for 2.5 minutes.

Supplementary Movie 2. Representative time-lapse video of dsRed-labelled mitochondria in axons of *MFN2*$^{R94Q/+}$ hESC-derived variant motor neurons. One image was taken every 500ms for 2.5 minutes.

Supplementary Tables

Supplementary Table S1: Quantitative mass spectrometry analysis of the endogenous MFN2 interactome in hESCs.

Tab A: Statistical analysis of MFN2 immunoprecipitations versus IgG controls across all genotypes.
Tab B: Bait-normalised statistical comparison of MFN2-associated proteins in *MFN2*$^{R94Q/R94Q}$ versus *MFN2*$^{+/+}$ cells.

Supplementary Table S2: Potential off-target CRISPR guide sequences

Sequence	PAM	#Mis-matches	Gene	Chromosome location
TCAGTGAGGTGCTGGCTCGG	AGG		MFN2	chr1:+11992641
CAAGTGAGGTGCTGGCTCGG	AGG	2		chr12:+6855936
TAAGGGAAGTGCTGGCTCTG	AGG	4		chr1:+14882608
TCAGAGAGGTGCTGACTCTG	AGG	3		chr5:-122518509
TCCTTGAGGTGCTGACTCTG	CAG	4		chr8:+76558285
TAAGTAAGCTGCTGACTCGG	TGG	4		chr9:+129111950

Mismatches are versus the guide used to generate the MFN2 R94Q containing lines

Supplementary Table S3: Table of Primers

Target	Forward Primer	Reverse Primer
GAPDH	agccacatcgctcagacac	gcccaatacgaccaaatcc
LHX1	atgcaacctgaccgagaagt	caggtcgctaggggagatg
BRACHYURY (TBXT)	tgacaaacaaccagctcttca	tgagggctcagcacttgtt
OLIG2	aggtacccaaccctgagga	gcaggtgagttgtcagaataggt
ISLET1	agctcctcaaatcgcatcc	atagtcgtcgcagctttcg
MNX1 (HB9)	gcagcccaatgacaaaactaa	ccgtcgtgtctctctggact
HOXA4	ttacctgacttatgaaacttgaaacc	cccagagacgtaagcataaacc
HOXB5	gttgccacccaagagagaac	ccaagtagtccttctcaggtatcc
HOXB6	aagcttcacatcagccatga	cggttgaagtggaactccttt
HOXB7	tggaagctgaagaagaaactgaa	gccgggtttatgatttgttg
HOXC8	ctacccctggatgcgaag	caggtagcgattgtagtgaaattct
HOXC9	tcctagcgtccaggtttcc	gctacagtccggcaccaa
FOXA2	tcctagcgtccaggtttcc	gctacagtccggcaccaa
PAX6	cgagatttcagagccccata	aagacaccaccgagctgatt
CHAT	cgaggagagcaggtccaca	ctgctgggagtttttgctgc
PHOX2B	ctaccccgacatctacactcg	ctcctgcttgcgaaacttg
NANOG	aactctccaacatcctgaacc	ccttctgcgtcacaccatt

Supplementary Table S4: Primary Antibodies for Immunofluorescence

Target	Company	Catalogue Number	Dilution
CHAT	Abcam	AB144P	1:100
ISLET1/2	DSHB	39.4D5	1:200

HB9	DSHB	81.5C10	1:50
FOXP1	R&D	MAB45341	1:150
NFH	Antibodies.com	A85336	1:500
Cleaved Caspase-3	Cell Signalling Technology	9661	1:400
Peripherin	Millipore	AB1530	1:100
Tuj1	Abcam	AB7807	1:200

Supplementary Table S5: Secondary Antibodies for Immunofluorescence

**Target**	**Company**	**Catalogue Number**	**Dilution**
Goat Secondary (594)	Invitrogen	A-11058	1:500
Mouse Secondary (488)	Invitrogen	A-21202	1:500
Rabbit Secondary (647)	Invitrogen	A-31573	1:500
Rabbit Secondary (594)	Invitrogen	A-21207	1:500

Supplementary Table S6: Primary Antibodies for Western blotting

**Target**	**Company**	**Catalogue Number**	**Dilution**
MFN2	CST	D2D10	1:1,000
MFN1	CST	D6E2S	1:1,000
Tubulin	Abcam	AB_2288001	1:10,000
Acetylated α-tubulin	Sigma	6-11B-1	1:5,000

GAPDH	CST	14C10	1:2,000
B-Actin	Proteintech	60008-1-Ig	1:10,000

Supplementary Table S7: Secondary Antibodies for Western Blotting

**Target**	**Company**	**Catalogue Number**	**Dilution**
Mouse Secondary (800)	Stratech	715-655-150-JIR	1:50,000
Rabbit Secondary (700)	Stratech	711-625-152-JIR	1:50,000
