# Supplementary material for: HDAC6 inhibition alleviates mitochondrial trafficking in models of Charcot-Marie-Tooth disease type 2A
Source: JCI Insight. 2026 Jul 14;11(16):e200106. doi: 10.1172/jci.insight.200106 (PMC13502185; doi:10.1172/jci.insight.200106)
Supplement: Supplementary file 2 [file jciinsight-11-200106-s132.pdf]

Full unedited gel for Figure 1C:

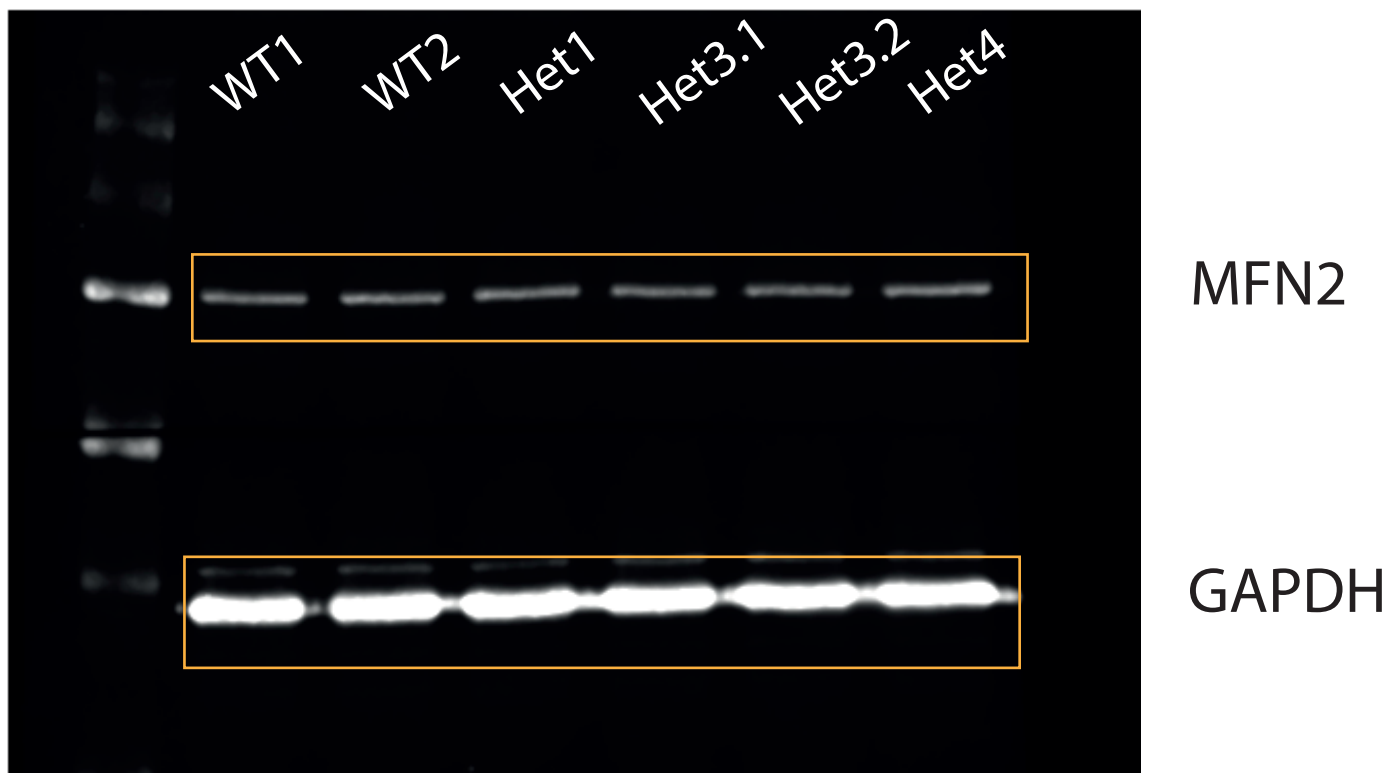

Full unedited gel for Figure 6A:

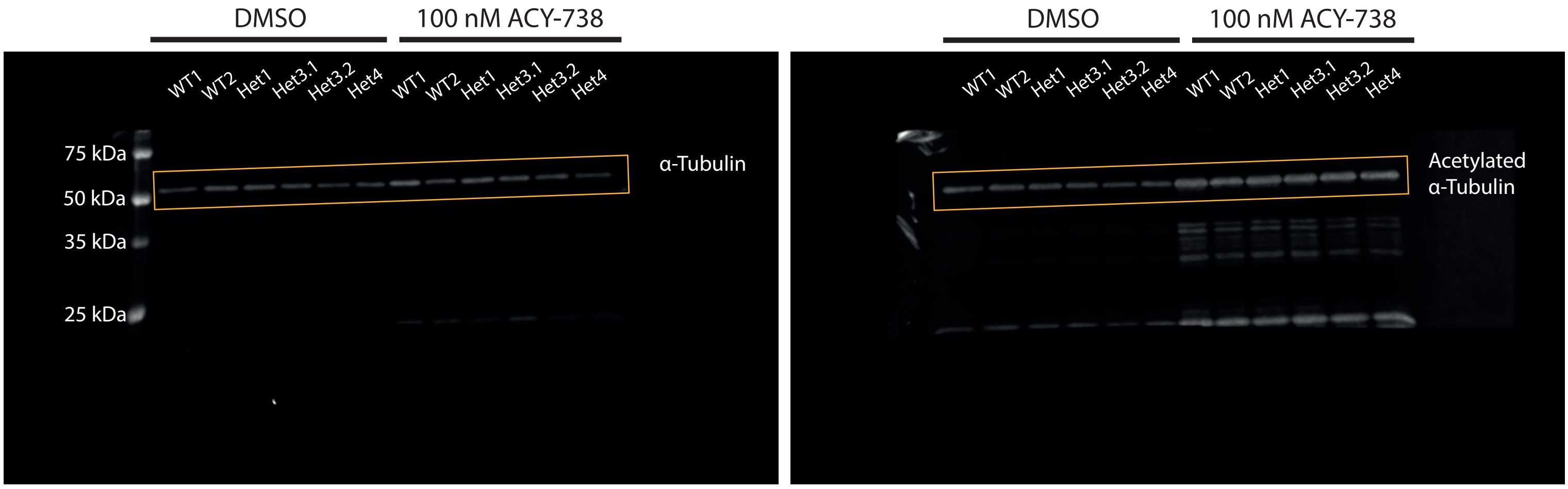

700 channel

800 channel

Full unedited gel for Supplementary Figure 13A:

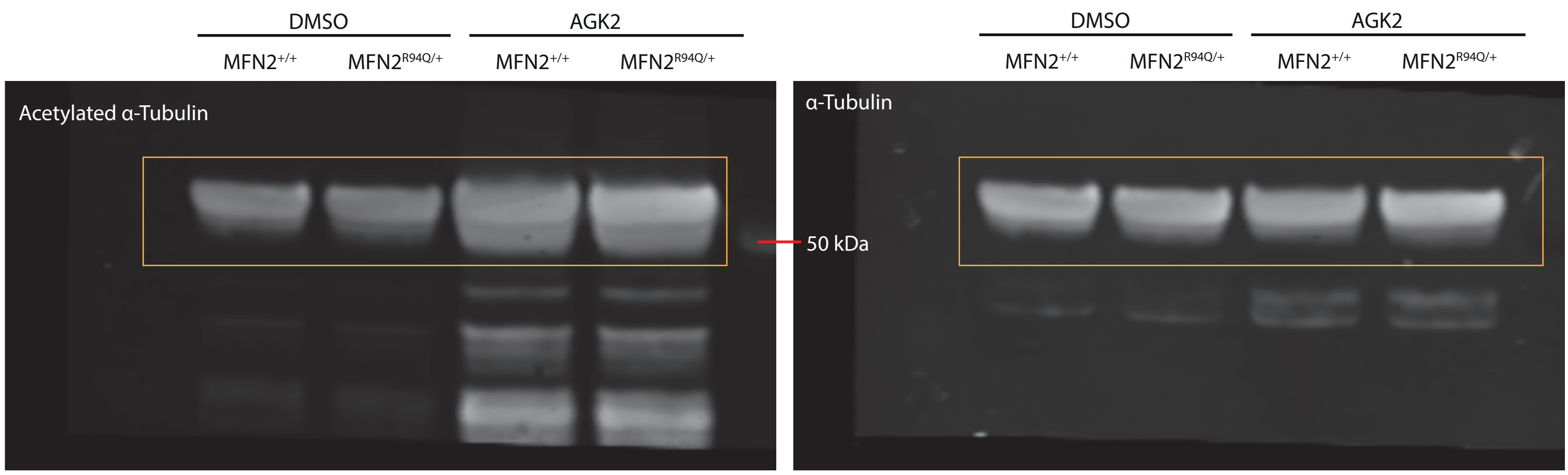

700 channel

800 channel
